# Continuity or change in the transition to Islam? A diachronic assessment of agricultural production at Old Dongola, Northern Sudan (14th–18th centuries CE)

**DOI:** 10.1371/journal.pone.0353303

**Published:** 2026-07-09

**Authors:** Mohammed Nasreldein, Simone Riehl, Artur Obłuski

**Affiliations:** 1 Institute for Archaeological Sciences, University of Tübingen, Tübingen, Germany; 2 Department of Archaeology, Faculty of Arts and Human Sciences, University of Gezira, Wad Madani, Sudan; 3 Senckenberg Centre for Human Evolution and Paleoenvironment, University of Tübingen, Tübingen, Germany; 4 Polish Centre of Mediterranean Archaeology, University of Warsaw, Warsaw, Poland; Maria Curie-Sklodowska University: Uniwersytet Marii Curie-Sklodowskiej, POLAND

## Abstract

In this paper, we present an archaeobotanical study from Old Dongola, northern Sudan, examining agricultural dynamics and subsistence strategies from the Late Christian to the Islamic period. Old Dongola, established in the 5th century, served as the capital of the Kingdom of Makuria, one of the three medieval Nubian kingdoms. Following the collapse of Makuria in the 14^th^ century, the city underwent significant cultural and social transformations, marked by the migration of Arab tribes and the rise of new power structures. From the 14^th^ to the 18^th^ century, Old Dongola served as the seat of a local ruler who became subordinate to the Funj Sultanate in the 16^th^ century, a period that witnessed religious conversion, migration, and political reorganisation associated with the gradual conversion to Islam in the region. This study is distinguished by a systematic sampling strategy, with sediments collected from every excavated context in the citadel of Old Dongola. The archaeobotanical results, spanning the Kingdom of Dongola and Funj periods (14^th^–18^th^ centuries), shed light on the dietary habits of the site's inhabitants. The evidence indicates an increasing reliance on sorghum, alongside a gradual decline of bread wheat and hulled barley. The introduction of crops such as tobacco and eggplant from the 16^th^ century onwards may reflect broader economic and cultural shifts occurring during this period of religious and demographic change, though a direct causal link between these crop introductions and processes of demographic change or conversion to Islam cannot be established from archaeobotanical evidence alone. This study contributes to our understanding of agricultural change in early modern Sudan, adding to an understudied archaeobotanical record at a time of significant socio-political transition.

## Introduction

For centuries, agriculture has formed the backbone of Nile Valley societies and continues to play a central role today, particularly in Nubia (in present-day northern Sudan). Alongside agriculture, practices such as animal husbandry, hunting, fishing, and trade collectively shaped the economic structure of ancient Nubian communities [1–5]. Previous studies have emphasised the consistency of staple economic patterns throughout Sudanese history, with only gradual shifts in response to environmental and demographic changes [6–10]. The Nile River has long been the lifeline of the region, supporting settlements along its banks, where seasonal flooding deposited fertile silt and created ideal conditions for cultivation [11–14]. In this context, agricultural seasonality was closely tied to the annual inundation of the Nile and the deposition of nutrient-rich silt, which created favourable conditions for crop production [12,15,16].

The arable lands along the riverbanks are limited to narrow strips of silt-rich soil, with the exception of basins such as those of Selim or Letti, where the fertile zones expand significantly. These broader floodplains supported key centers of political and economic life in different periods: for instance, the Selim basin was integral to the rise of the first Kingdom of Kush around 2500 BCE [17–19], while the Letti basin contributed to the agricultural foundation of the Makurian Kingdom from the 5th century CE onward [20]. Despite the harsh terrain, extreme heat, and periods of prolonged drought, the ancient Sudanese populations developed localized and adaptive strategies to make this environment productive [6,15,19,21,22]. These strategies, adapted to floods and drought periods, included an agricultural calendar that structured the rhythms of planting and harvesting, as well as the adoption of irrigation technologies like the *saqiya* – the Persian waterwheel – to expand the arable lands [11,23].

However, the archaeobotanical evidence from this region remains uneven [24–27], despite a broader archaeological record, largely because archaeobotanical analysis has only recently become a research priority and remains limited in scope and publication. Much of the current understanding of ancient Sudanese agriculture relies on fragmented studies, often limited in spatial or chronological scope. The largest and most comprehensive dataset for the Nubian region in general comes from Qasr Ibrim in southern Egypt; a uniquely preserved site that spans the Late Pharaonic to Islamic periods and provides valuable diachronic evidence for agricultural change [27]. Other studies offer region-wide overviews but rarely focus in detail on the post-medieval period [24,25,28,29]. However, medieval and early Arabic sources (e.g., al-Maqrīzī, Ibn Salim al-Uswānī) complement the archaeobotanical evidence, offering insights into the importance of crops like wheat, barley, dates, and sorghum during this time [2,30,31].

**Fig 1:**
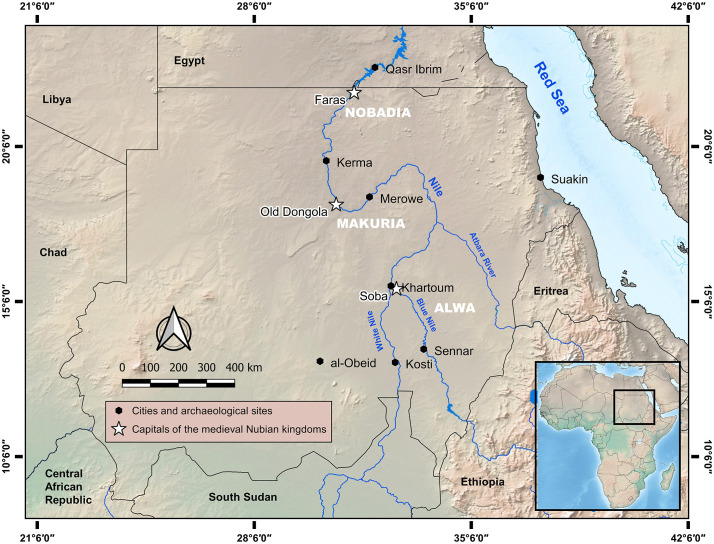
General map of Sudan showing the location of Old Dongola and the other capitals of medieval Nubian Kingdoms (prepared by Mohammed Nasreldein). Vector data provided by Natural Earth.

The available literature on diachronic changes in agricultural production suggests a shift in farming practices during the Meroitic kingdom (350 BC–350 CE), often linked to the introduction of the Persian waterwheel (*saqiya* in Arabic) [1,2,5]. Nevertheless, archaeological evidence for its widespread use at this time remains limited, and its large-scale adoption, together with the expansion of arable land and population growth, appears to have occurred later, during the Three Kingdoms period (Nobadia, Makuria, Alwa) in the sixth to eighth centuries CE (Fig 1). According to Fuller [25], this innovation marked a turning point for Nubian agriculture by enabling the expansion of arable lands along the riverbanks, islands, and adjacent lands from the river, increasing harvest yields, and facilitating cultivation during both winter and summer seasons.

Thus, the *saqiya* is generally understood to have enabled the expansion of cultivation into both winter and summer seasons, potentially supporting double-cropping agriculture, which became key components of the ancient Sudanese economy, particularly during the Meroitic period [24,32,33]. Sorghum, in particular, emerged as a dominant summer crop and a staple food crop of the ancient Sudanese diet [34–38]. Previously, farming was largely confined to narrow silt-rich strips along the riverbanks after the flood season, which were linked to the so-called *garf* and *seluka* lands – *seluka* being a wooden stick that the Nubians used to make holes for sowing seeds along sloping riverbanks and islands [23,39].

Previous studies indicate that after the introduction of *saqiya*, alongside the expansion of cotton and sorghum cultivation, other crops like wheat and barley continued to be grown, albeit with less prominence and varying levels of importance across different regions [13,25,40–42]. Furthermore, no major shifts in agricultural practices were recorded until the Ottoman incursion into northern Sudan in the 16th century, when crops from the New World were introduced [25,26]. A later transformation came with the gradual replacement of traditional irrigation methods with mechanical water pumps, and the increasing use of chemical fertilisers in place of organic ones, starting after the 1900s [11,12,14,43]. Ethnographic studies also note the gradual abandonment of the Coptic agricultural calendar, which is giving way to the Gregorian calendar as Nubian farming practices become increasingly aligned with national agricultural cycles coordinated by government bodies and agricultural institutions [23]. Beyond these developments, prior studies do not point to any substantial or radical changes in Nubian agricultural practices.

**Fig 2:**
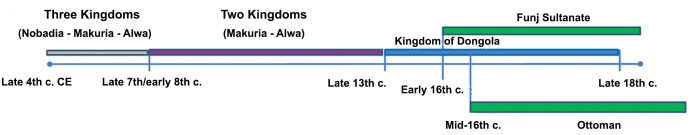
Chronology of medieval and post-medieval Nubia, after Obłuski [44].

Based on our recent archaeobotanical investigations at Old Dongola, in this paper we try to answer the question of how local Nubian agricultural practices and crops have changed over time, particularly in the 14th-18th centuries CE, a period that witnessed the collapse of the Christian Nubian kingdom of Makuria in the 14^th^ century (Fig 2). Thereafter, the city underwent significant cultural and social transformations marked by the migration of Arab tribes and the rise of new power structures. During the 16^th^ and 18^th^ centuries, Old Dongola served as the seat of a local ruler under the Funj Sultanate (1504–1821 CE), reflecting its integration into wider Islamic governance. During this period, diverse communities emerged as a result of religious conversion, migration, and broader political change. These developments shaped the long-term occupation of the city, which continued into the colonial era, and reflect its complex historical trajectory. In light of these substantial social and political transformations within Nubian society, this study explores whether such changes are visible in the archaeobotanical assemblage and examines their implications for agricultural practices and crop production. By doing so, our research addresses an important gap in current knowledge and contributes to local and regional discussions of agricultural production and dietary change.

### The archaeological site

**Fig 3:**
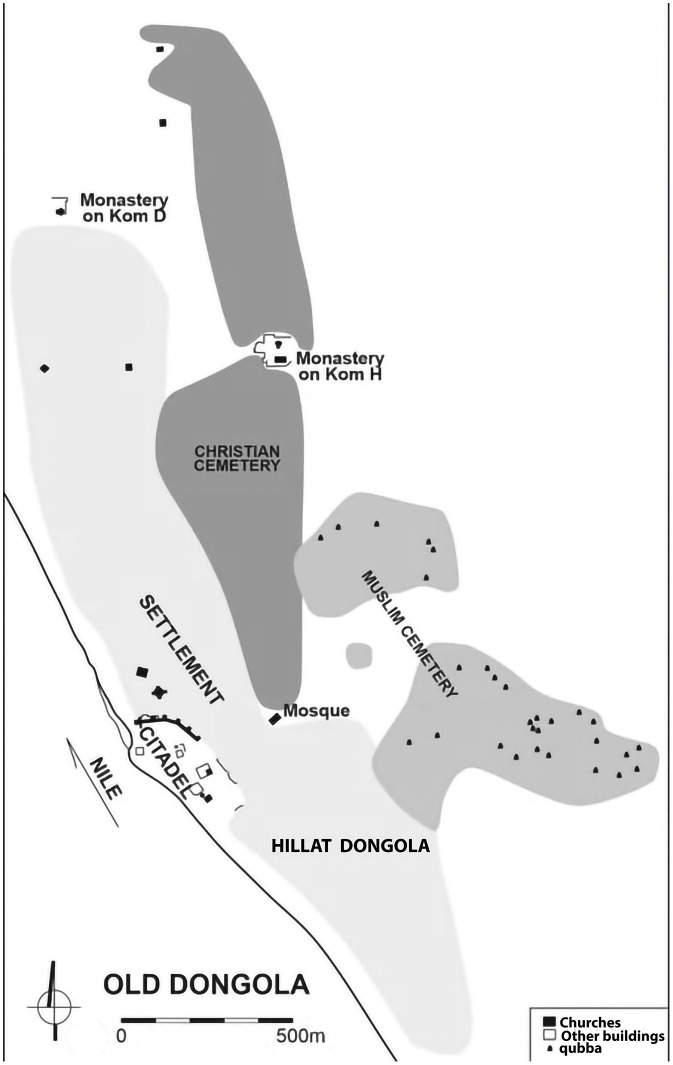
Archaeological map of Old Dongola (drawn by Agata Deptuła and Szymon Maslak). Reprinted from [46] under a CC BY 4.0 license, with permission from the Polish Centre of Mediterranean Archaeology, University of Warsaw (PCMA UW), original copyright (2026). Courtesy of the Polish Centre of Mediterranean Archaeology, University of Warsaw.

Old Dongola (Old Nubian *Tungul*, Arabic *Dunqula* - *دنقلا*) is located in Sudan's Northern State province (N 18.1323°, E 30.4438°). The site was uniquely positioned for agricultural production, being situated on the eastern Nile riverbank on the southern margin of the Letti basin, which was formed by a Nile paleochannel [20,45,46]. The location of Old Dongola at the end of the Wadi Howar, an important Sub-Saharan communication route, facilitated its growth [46]. According to Godlewski [20], the city was founded in the 5^th^ century by one of the first kings of Makuria. Old Dongola became the capital of Makuria (500–1300 CE), one of the three medieval Nubian kingdoms [5,47]. As the city developed, its size reached approximately 200 ha (Fig 3) in its heyday between the 10^th^ and 12^th^ centuries [46]. At the end of the 13^th^ century, following the Mamluk takeover of Egypt, the regional political situation deteriorated, culminating in the 1276 invasion during which the Makurian state was subdued and became a vassal of Egypt [31]. According to the Arabic sources, the Makurian kings relinquished parts of the territory to the sultan, paid an annual tribute, and later imposed a *jizya* tax on Christians (a tax historically levied on non-Muslim subjects under Islamic rule as a form of tribute or protection tax) [31].

Although historical sources continued to refer to the kings of Makuria as local governors, Arabic sources gradually replaced the name Makuria with the Kingdom of Dongola, as Makurian elites resisted Mamluk control for nearly a century [44]. Mamluk interest in Makuria gradually declined, and around the mid-14^th^ century, the situation became more complex with the migration of various Arabs tribes into the Middle Nile Valley [48]. During the Funj Period (1504–1821 CE), the kingdom of Dongola was an essential part of the Funj Sultanate and was often involved in internal power struggles [44]. Following the disintegration of the Funj Sultanate in the 18^th^ century, the kingdom of Dongola fell to the *shaiqiya* invasions, with the last king of Dongola ceasing to use the royal title in the 1780s [44].

Archaeological work in Old Dongola began in 1964 by the Polish Centre of Mediterranean Archaeology of the University of Warsaw [20]. In 2018, a new phase of archaeological investigations began at Old Dongola, marking a renewed focus on urban and social transformations in the later periods of the city’s history. One of the main objectives of the project is to investigate the transformation of Old Dongola from a Christian community into a new socio-political entity between the 14^th^-15^th^ centuries, as well as the impact of the migration of Arab tribes on the social structure of the inhabitants after the collapse of the Makurian kingdom [46,49,50]. Despite continuous research efforts on the site since 1964, a comprehensive understanding of the cultural dimensions surrounding subsistence regimes and cash crops has remained elusive. To address this gap, we conducted extensive, large-scale archaeobotanical sampling within the citadel of Old Dongola, aiming to unravel the intricacies of local agricultural production and trade and to reconstruct the subsistence patterns at Old Dongola.

### Agriculture and irrigation in the Dongola Reach: the case of the Letti basin

**Fig 4:**
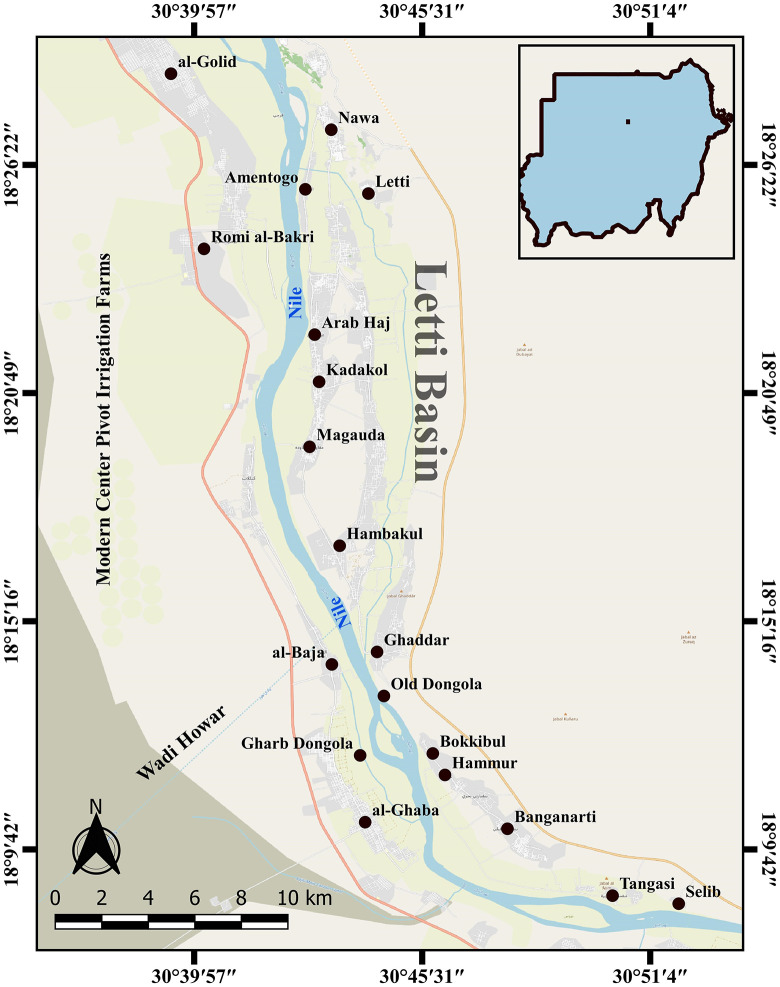
The Letti basin and surrounding towns, including Old Dongola (prepared by Mohammed Nasreldein). Vector data provided by OpenStreetMap.

The Dongola Reach, stretching between the Third and Fourth Cataracts and covering c. 500 km, has long held agricultural and economic importance in Nubia [11,12,39]. Areas such as the Letti basin (Fig 4), Kerma and Merowe (Fig 1) were historically densely populated, benefiting from nutrient-rich alluvial soils left by annual Nile floods [18,19,47]. The region lies within a hyper-arid zone with minimal rainfall and extreme heat [51,52], conditions that generally restrict vegetation to riverbanks and seasonal watercourses [53]. Recent extreme weather, such as the 2024 flash floods in the Dongola Reach as witnessed during fieldwork, reflects increasing climatic unpredictability. These observations highlight the complex relationship between soil quality, irrigation timing, and agricultural productivity in the Letti basin. While certain sections (*gism*) had promising agricultural potential, others were severely limited by high sand content, making large-scale irrigation efforts economically unfeasible without significant infrastructural investments. Agriculture in the Letti basin remains an understudied topic, with many aspects of historical land use, water management, and agricultural adaptation still requiring further research.

The Letti basin (Fig 4) populations combined two key farming methods, integrating seasonal flood-based farming with early irrigation. The first, *garf* cultivation – one of the earliest farming strategies in the region – involved narrow and fertile strips along riverbanks dependent on flood recession [1,12,23]. The second, the hod system, relied on shallow basins to trap floodwater and silt [11,16,54]. However, both systems relied on natural inundation, making productivity inconsistent. The introduction of water-lifting devices, such as the *shadoof* and later the *saqiya*, transformed irrigation practices in the region. The *shadoof*, a simple counterpoise lift operated by manpower introduced to the region during the New Kingdom (c. 1500–1000 BCE) [1,11], allowed for manual water retrieval from the river, but its reach was limited. The *saqiya*, a waterwheel mechanism introduced during the Meroitic period (c. 350 BC – 350 CE), was a significant innovation that enabled water to be lifted to higher elevations, extending the cultivation zone beyond the immediate floodplain and enabling year-round farming, reducing reliance on seasonal floods [1,5,11,25].

A particularly distinctive feature of irrigation in the Dongola Reach was the adaptation of the *saqiya* for use with wells, known locally as *matara* [12,55,56]. This innovation meant that even areas at a greater distance from the river could be cultivated, mitigating the limitations of Nile-dependent farming [56–58]. Historical accounts from the 19^th^ century confirm that this *matara*-and-*saqiya* system was still in use in the Dongola Reach, with reports indicating that lands in the Kerma and Letti basins were cultivated using wells located more than half a mile from the riverbank [59]. Archaeological evidence further supports the existence of this method at Letti basin during the Meroitic period [60]. Additionally, notable discoveries of well-preserved *matara*-and-*saqiya* structures were recorded near Old Dongola, close to a church site at Selib (for the location of Selib see Fig 4), and close to a Meroitic settlement site, which lies almost a kilometre away from the Nile [61]. The economic history of Nubia suggests that while farming provided essential food resources, local elites did not rely on agricultural surplus for wealth, instead; they maintained power by controlling strategic trade routes and local resources [7,9,62–65].

A 1912 British report divided Letti into four sections (*gism)* based on soil fertility, further subdivided into *hods* and *feddan* (4200 m²) plots (*tarabeel*) [66]. Only 3/8 of the 83,000 *feddans* were cultivable. Crop selection was linked to silt depth: for instance, termis (*Lupinus albus* L*.*) required 10 cm, barley 30–50 cm, and wheat over 70 cm. Irrigation followed a 50-day filling and 10–15-day drainage cycle, with sowing starting late October. Although this report provides valuable technical insight, it reflects colonial planning logic and calls for renewed investigation within the broader environmental and historical context.

## Materials and methods

**Fig 5:**
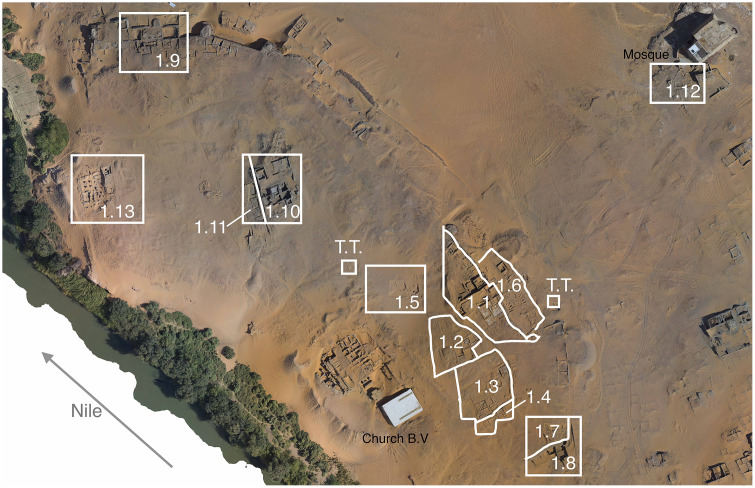
An orthophoto map of the citadel of Old Dongola, showing the entire excavated zones of the project (Prepared by Adrian Chlebowski). Obtained under a CC BY 4.0 license, with permission from the Polish Centre of Mediterranean Archaeology, University of Warsaw (PCMA UW), original copyright (2026). Courtesy of the Polish Centre of Mediterranean Archaeology, University of Warsaw.

**Fig 6:**
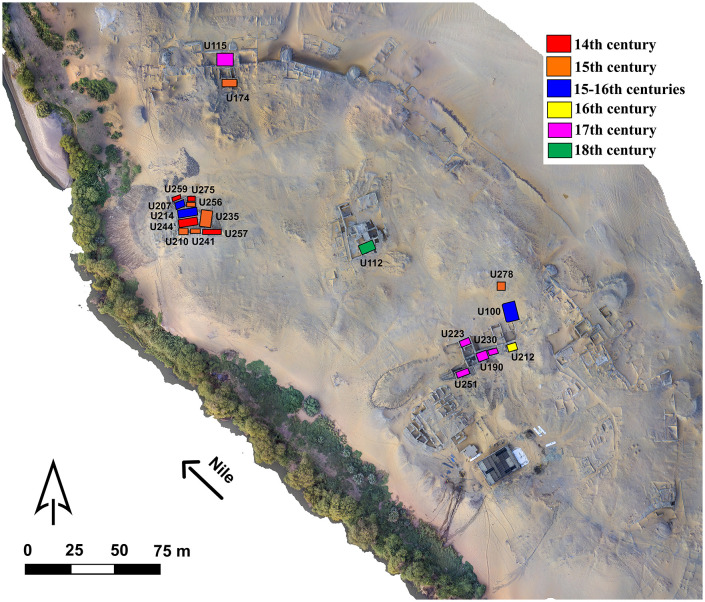
An orthophoto map of the citadel of Old Dongola, highlighting the excavation units in which sediment samples were collected for this study (Prepared by Adrian Chlebowski and Mohammed Nasreldein). Obtained under a CC BY 4.0 license, with permission from the Polish Centre of Mediterranean Archaeology, University of Warsaw (PCMA UW), original copyright (2026). Courtesy of the Polish Centre of Mediterranean Archaeology, University of Warsaw.

This study examines 44 sediment samples (totalling 515 litres of sediment) collected from the citadel of Old Dongola during excavation seasons from 2018 to 2023 (Fig 5 and Fig 6). The samples were systematically collected from every excavated context for archaeobotanical analysis, with volumes ranging from 1 to 20 litres per sample [46,67]. The selection criteria prioritized contexts rich in organic material and directly associated with human activities, including animal dung accumulations, hearths, middens, and sandy soils with organic matter, likely representing activity areas. Archaeological contexts were defined during excavation based on stratigraphic and architectural criteria and are supported by previous high-resolution analysis integrating geochemical, spatial, and botanical data [68]. In addition, all necessary permits for excavation, sampling, and analysis were obtained from the National Corporation for Antiquities and Museums (NCAM), Sudan.

Detailed information on each sampled context, including context type, excavation unit, and sample characteristics, is provided in Supplementary S1 Table. The site is located in an arid environment characterized by hot and dry conditions, which favour the preservation of desiccated plant remains by limiting biological degradation (see Section “Agriculture and irrigation in the Dongola Reach: the case of Letti basin” for environmental context). Although most contexts are not sealed, sampling focused exclusively on defined archaeological contexts associated with human activity and was carried out under controlled conditions to minimize contamination. The recovered plant remains show clear signs of desiccation, suggesting their preservation is primarily due to local environmental conditions rather than recent intrusion (see also Nasreldein [69] for a detailed discussion). However, since no external control samples were collected from non-archaeological contexts, the possibility of later intrusions of domesticated or wild plant material cannot be entirely excluded, and this limitation is considered in the interpretation of the assemblage. The chronological attribution of the samples is based on radiocarbon-dated archaeological contexts, using other organic materials recovered during excavation.

Sediment processing took place during the 2022−23 field seasons using a combination of water flotation and dry sieving methods [70,71]. Flotation was applied to the majority of sediment samples, while dry sieving was used selectively for samples derived from storage vessels or baskets. Flotation was performed using a series of metal mesh sieves (2 mm, 1 mm, 0.5 mm, and 0.2 mm), which were selected based on established archaeobotanical standards [71]. Each sample underwent three to four flotation cycles, with the buoyant fraction collected and transferred to fine nets (0.2 mm) for air drying. Subsequently, a 1 mm mesh size was used for air-drying the heavy fractions remaining in the bucket's bottom. After that, all the fractions were labelled and packed for transport. Although the sediments were largely dry and desiccated, flotation was necessary due to the presence of compacted deposits rich in organic material (e.g., dung), which hindered separation by dry sieving alone. No visible loss of macroremains was observed during processing.

The samples were further processed by sorting in the archaeobotany laboratory of the Institute of Archaeological Sciences, University of Tübingen, Germany. Before sorting, each sample was sieved through four nested geological screens (2 mm, 1 mm, 0.5 mm, and 0.2 mm) to facilitate the separation of botanical remains by size. The sorting strategy varied based on original sediment volume, following an initial assessment of sample richness during the sorting of early samples, which showed that larger samples were consistently rich in botanical remains. Accordingly, 1–3 litre samples were fully sorted, 3–10 litre samples were sorted by half, and samples exceeding 10 litres were sorted to one-quarter of their original volume. For partially sorted samples, the riffle-type sample splitter was used for subsampling. The final split flotation samples were packed for further examination. All counts reported in this study represent the actual number of remains identified from the examined material, without extrapolation or multiplication. Charcoal fragments were commonly observed in many samples. However, charcoal remains were not systematically quantified or analyzed in this study, which focuses on seed and fruit macroremains.

The identification of the macrobotanical remains was conducted through morphological and anatomical comparison with modern reference collections housed in the archaeobotany laboratory at the Institute of Archaeological Sciences, University of Tübingen. Additionally, we were able to access the herbarium specimens (particularly the African taxa) of the Botanical Garden at the University of Tübingen, aiding in the taxonomic identification of our archaeological materials. Further comparative analysis was conducted using established seed atlases and other botanical literature [72–77], which provided high-resolution reference data for seed identification. When taxonomic identification remained uncertain, specimens were classified at the genus level.

To refine taxonomic identifications, we used the African Plant Database (https://africanplantdatabase.ch/) to verify plant species distributions within Africa, particularly their natural habitats and presence in Sudan. Additionally, botanical nomenclature was cross-checked using *Plants of the World Online* [78], a taxonomic database maintained by the Royal Botanic Gardens – Kew, ensuring consistency with accepted scientific classifications, synonyms, and updated distribution records.

Correspondence Analysis (CA) was conducted using Canoco 5 (Windows release 5.04) [79] to explore general patterns of similarity in plant assemblages across samples. The aim of this analysis is exploratory, providing a visual assessment of relationships between samples and taxa rather than testing specific hypotheses. To prepare the data (S4 and S5 Files), samples containing fewer than 10 taxa (4 out of 44) were excluded to minimize potential outliers. Additionally, taxa present in fewer than 20% of the total samples (12 taxa) were removed to reduce the influence of rare occurrences and outliers in the ordination plots. Data was imported from Excel, ensuring taxa were in columns and samples in rows, with appropriate naming conventions for taxa and sample IDs. The CA analysis was set up via the Analysis Setup Wizard, selecting CA without detrending. Axes were chosen based on eigenvalues and cumulative explained variance, and default settings were adjusted to avoid limiting the number of taxa or samples displayed. The workflow followed guidelines from Vermeersch et al. [80], including the creation of classifications for samples (or species) within Canoco 5. Classes were defined via the Project > Classifications menu, assigned manually using the “By Selection” option, renamed as needed, and activated for graphing. Biplots were updated via the re-create graph function. Final Figures were refined using graphic editing software (e.g., Inkscape, Photoshop) for clarity and publication purposes.

Additional information regarding the ethical, cultural, and scientific considerations specific to inclusivity in global research is included in the Supporting Information (S6 File).

## Results

The assemblage consists of both desiccated and charred plant remains, with desiccated specimens predominating. This preservation pattern reflects the arid environmental conditions of the site, which are known to favour the survival of uncharred plant material. Although most contexts are not sealed, sampling was carried out under controlled conditions to minimize contamination, and the recovered remains show clear signs of desiccation consistent with *in-situ* preservation. However, since no external control samples were collected from non-archaeological contexts, the possibility of later intrusions, whether domesticated or wild species, cannot be entirely excluded. This limitation is considered in the interpretation of the assemblage and claims regarding crop presence and dietary patterns are made with this point in mind. The co-occurrence of charred and desiccated remains in some contexts likely reflects a combination of activities, including food preparation, fuel use, and the burning of dung, and these different preservation pathways are taken into account throughout the discussion (see also Nasreldein [69] for a detailed discussion of preservation conditions at the site).

**Fig 7:**
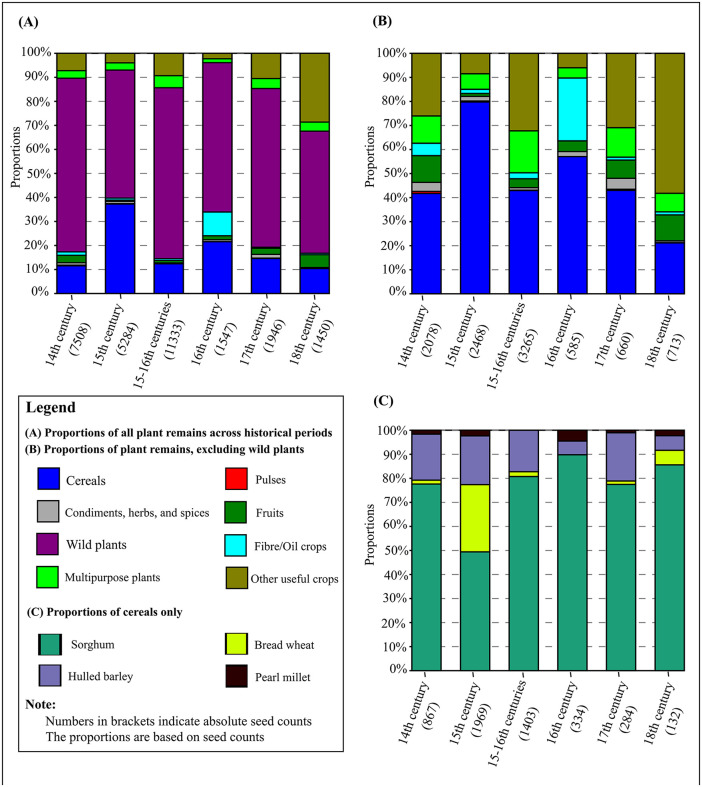
Proportional overview of plant remains grouped into major taxonomic categories, as defined in S2. Table. (A) Proportions of all plant categories; (B) Proportions excluding the wild plant category; (C) Proportions of cereals only. Numbers in brackets indicate the absolute seed counts for each historical period. (Prepared by Mohammed Nasreldein).

**Fig 8:**
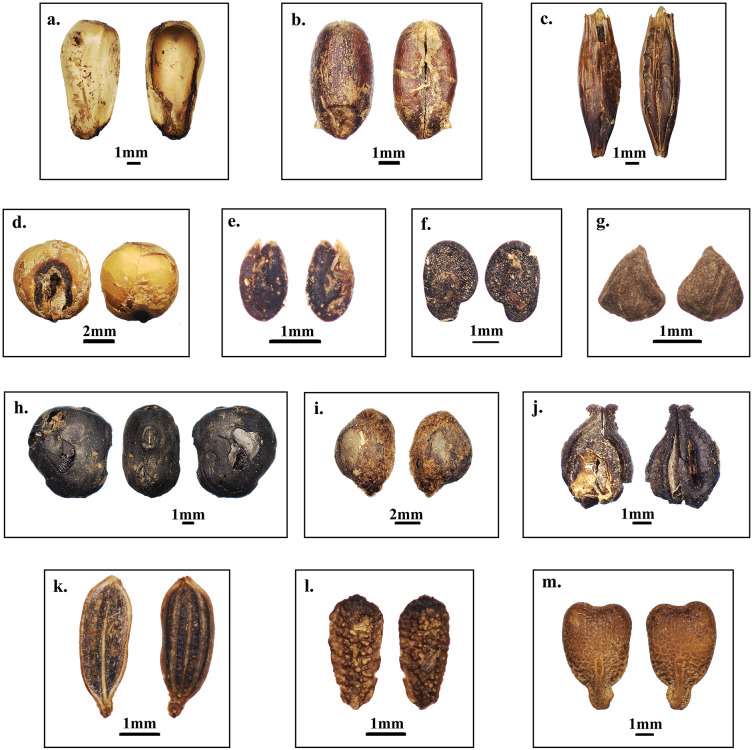
Representative examples of key crops discussed in this study, illustrating continuity and change in agricultural production at Old Dongola. All macroremains were preserved by desiccation, except for (h.) *Lupinus albus* (termis) seeds. a. Safflower (*Carthamus tinctorius* L.); b. Bread wheat (*Triticum aestivum* L.); c. Hulled barley (*Hordeum vulgare* L.); d. Sorghum (*Sorghum bicolor* (L.) Moench); e. Cress (*Lepidium sativum* L.); f. Eggplant (*Solanum melongena* L.); g. Jute (*Corchorus olitorius* L.); h. Termis (*Lupinus albus* L.); a. Cotton (*Gossypium* sp.); j. Grape (*Vitis* sp.); k. Anise (*Pimpinella anisum* L.); l. False daisy (*Eclipta prostrata* L.); m. Senna (Senna sp.). (Photos by Mohammed Nasreldein).

In total, 29,068 desiccated and charred seed remains belonging to 66 taxa were recovered (see S1 Table for preservation details and S2 Table for taxa), of which 22,960 (79%) are wild plant species (42 taxa), and 6108 (21%) seed remains of cultivated plants (24 taxa). These results indicate the dominance of the wild species in the assemblage over the cultivated plants (Fig 7A). After excluding the wild species as shown in Fig 7B, the cereals – which are represented by *Sorghum bicolor* (L.) Moench, *Triticum aestivum* L., *Hordeum vulgare* L., and *Pennisetum glaucum* (L.) R.Br. – dominates the assemblage, followed by different plant species that we categorised as multipurpose plants, including *Citrullus colocynthis* (L.) Schrad*., Hyoscyamus muticus* L., *Cleome* cf. *gynandra* L., *Malva* sp., *Lagenaria* cf. *siceraria, Sorghum halepense* (L.) Pers., and *Eclipta prostrata* L. After this was the category of the other useful crops; including *Nicotiana tabacum* L., *Solanum melongena* L., *Portulaca oleracea* L*., Corchorus olitorius* L. Additionally, during the 16^th^ century (Fig 7B), fibre and oil crops notably are represented, which is attributed to the high quantity of safflower (*Carthamus tinctorius* L.) desiccated seed remains (Fig 8A). However, this pattern is based on two samples, with the majority of remains deriving from a single context, and should therefore be interpreted with caution (see S2 Table).

**Fig 9:**
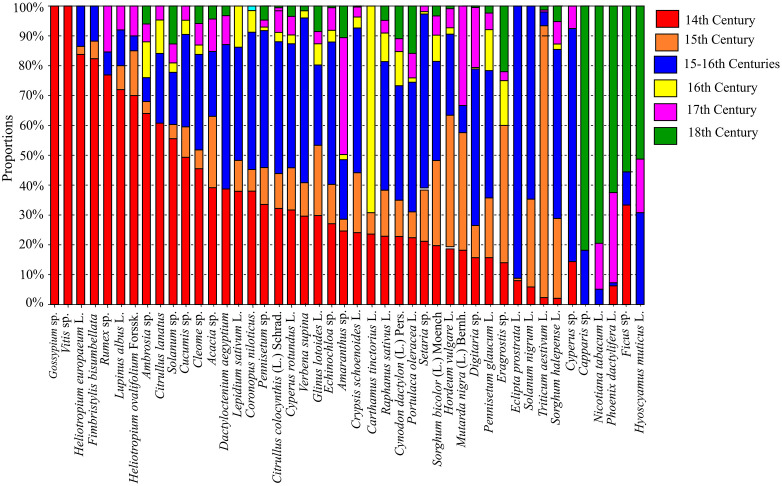
Proportional overview of the 45 most frequently occurring taxa in the archaeobotanical assemblage. Each colour represents a different historical period, illustrating changes in taxonomic representation over time. The Figure highlights diachronic patterns in plant use and the relative abundance of key taxa across the studied sequence.

This paper presents archaeobotanical data from five chronological phases at Old Dongola (Fig 9), beginning with the 14^th^ century – a transitional period marked by the decline of the Christian Kingdom of Makuria and the gradual rise of Islamic rule under the emerging Kingdom of Dongola. The dataset continues through the 15^th^, 16^th^, 17^th^, and 18^th^ centuries. It is necessary to clarify that within our sample set, some contexts date to the time bridging the second half of the 15^th^ century and the first half of the 16^th^ century. These were grouped as “15^th^–16^th^ centuries” in (S2 Table, Fig 6, Fig 7, and Fig 9), which includes 14 samples. Additionally, we have 6 samples dated to the 15^th^ century and 2 from the 16^th^ century. This clarification is necessary to avoid potential misinterpretations regarding the scope and representativeness of our dataset across time. In the following section, we present the archaeobotanical results for each century separately, beginning with the 14^th^ century. Each phase is discussed in terms of dominant cultivated and wild species, sample composition, and notable patterns in crop processing or use. Wild species are present throughout all chronological phases of the assemblage, showing a relatively stable pattern across the centuries with only minor variations in quantity. The composition of wild taxa does not vary dramatically and thus is not discussed in detail within each time frame unless a notable shift is observed.

### Period 1: 14th century

A total of 7,508 plant remains were recovered from samples dated to the 14^th^ century. The overall assemblage from this period was dominated by wild species, which comprised 76% of the total, while cultivated plants accounted for 24%. This phase stands out within the broader dataset, as it yielded certain taxa that were either unique to this period or are only rarely attested elsewhere in Sudan. Notable examples include grape (*Vitis* sp.), anise (*Pimpinella anisum* L.), cotton, (*Gossypium* sp.), mallow (*Malva* sp.), and the wild jute (*Corchorus olitorius* L). (Fig 8). These taxa were identified only in the 14^th^-century assemblage and do not appear in samples from subsequent periods (see S2. Table), suggesting a phase-specific presence. In terms of cereal consumption, the assemblage from the 14^th^ century does not reflect a significant shift from the patterns observed in later periods. However, sorghum is already well-represented in this phase, while bread wheat and hulled barley are present in notable quantities. Most findings of termis (*Lupinus albus* L.) in our assemblage were also recovered from this period. One of the interesting findings from this period is the discovery of false daisy (*Eclipta prostrata* L.), which we only encountered during this period and the following two centuries (15^th^ and 16^th^). Aside from these examples, no major changes in plant consumption practices were observed.

### Period 2: 15th century

Samples from this period (6 samples) yielded a total of 5,284 plant remains. The assemblage is dominated by wild species, which represent 57% of the total, while cultivated crops account for 43%. Compared to the previous period, and despite the smaller number of samples analyzed, we observed an increase in cereal remains – particularly bread wheat and hulled barley. Sorghum also shows an increase, and notably, this period produced the largest quantity of pearl millet recovered in the entire assemblage (see S2. Table; Fig 7B). Traces of legumes were also identified, most notably cowpea (*Vigna unguiculata* (L.) Walp.), which is rarely attested in the broader assemblage. Other noteworthy discoveries from this period include evidence of carob (*Ceratonia siliqua* L.) and bottle gourd (*Lagenaria* cf. *siceraria*), neither of which appear in assemblages from other periods. In addition, coriander (*Coriandrum sativum* L.) was identified among the remains; interestingly, coriander appears only in the samples dating to the 14^th^, 15^th^, and 16^th^ centuries.

### Period 3: 15th-16th centuries

Most of the samples (14 in total) analyzed in this study originate from contexts dated to the transitional period between the second half of the 15^th^ century and the first half of the 16^th^ century. These samples yielded a total of 11,333 plant remains, of which 76% were wild species, while cultivated plants accounted for 24%. This period is marked by a noticeable increase in sorghum remains and a reduction in bread wheat, while hulled barley maintains a consistent presence. Notably, no remains of pearl millet were encountered in this period. Among the rarest finds in the entire dataset is the seeds of African peach fruit (*Nauclea latifolia* Sm.), which first appeared in this period and continues to be present in the later centuries. Additionally, two crops of particular interest also appear in this assemblage: tobacco (*Nicotiana tabacum* L.) and eggplant (*Solanum melongena* L.). Both persisted in the following centuries, although eggplant was absent again until the 18^th^ century. The tobacco remains were recovered from an occupational layer (Context 1185 S.2743) dated by radiocarbon between the fourth quarter of the 15^th^ century and the third quarter of the 16^th^ century (1476–1575 CE). Another notable find from this period is the first appearance of Egyptian henbane (*Hyoscyamus muticus* L.), a plant identified for the first time in the region, which continues to appear in the assemblages of the following centuries. This period also marks the final appearance of black nightshade (*Solanum nigrum* L.), which had been previously recorded in the 14^th^ and 15^th^ centuries but is absent from the assemblages of later periods. Aside from these patterns, no major changes in plant use are observed during this phase.

### Period 4: 16th century

Samples dated to this period yielded a total of 1,547 plant remains, of which 64% were wild species and 36% cultivated plants. Sorghum continues to be present in this phase, while bread wheat is notably absent. Hulled barley shows a significant decrease, and pearl millet reappears after its absence in the previous period. This period also marks the final appearance of cress (*Lepidium sativum* L.) in the assemblage; it had been identified in contexts ranging from the 14^th^ century up to this point but is absent in later phases. One particularly interesting find is the presence of senna (*Senna* sp.) (Fig 8M), which represents the only occurrence of this plant across the entire assemblage. Additionally, the largest quantity of safflower (*Carthamus tinctorius* L.) remains was recovered from this period – a total of 135 desiccated seeds from a single sample – which might indicate an intensive use of this plant at this time. Aside from these observations, no other notable changes were recorded during this period.

### Period 5: 17th century

A total of 1,946 plant remains were identified from six samples dated to this period, of which 71% were wild species and 29% cultivated plants. In terms of cereal consumption, sorghum continues to feature prominently, with hulled barley and bread wheat also present, although in noticeably smaller quantities than in earlier centuries – indicating a continued decline in their cultivation. Pearl millet also persists in this period. One of the few noteworthy additions is the appearance of a rare wild species, cinquefoils (*Potentilla supina* L.), which also occurs in the subsequent century. Beyond this, the overall pattern of plant use remains largely consistent with that of the 16^th^ and 18^th^ centuries, with no major shifts observed.

### Period 6: 18th century

The assemblage from this period, represented by four samples, yielded 1,450 plant remains, of which 56% were wild species and 44% cultivated crops. Sorghum and pearl millet continue to show a strong presence, while bread wheat and hulled barley appear in reduced quantities compared to earlier periods. This phase is marked by a notable absence of condiments, herbs, and spices that were more frequently represented in the previous centuries. In terms of fruit consumption, it is particularly striking that cucumber/melon (*Cucumis melo/sativus*), which was present consistently in all previous assemblages, is entirely absent in this period. Conversely, the majority of finds of the wild fruit *karir* (*Capparis* cf. *decidua*) come from this phase. This period also yielded the highest number of Egyptian henbane (*Hyoscyamus muticus* L.) remains across the assemblage, along with an increased number of tobacco (*Nicotiana tabacum* L.) finds. Aside from these patterns, no further significant changes in plant use were observed.

In sum, the archaeobotanical assemblages from Old Dongola reflect both continuity and change in plant use between the 14^th^ and 18^th^ centuries CE. Sorghum remains dominant throughout, while bread wheat and hulled barley slightly decline over time. Certain species – such as coriander and black nightshade – disappear entirely in later periods, while others, including Egyptian henbane and tobacco, appear for the first time and continue into subsequent centuries. Although some phases show minimal change, others highlight notable shifts in crop composition and plant use, providing a basis for broader reflections on cultural and environmental dynamics, which are addressed in the discussion.

### Correspondence analysis of plant assemblage

**Fig 10:**
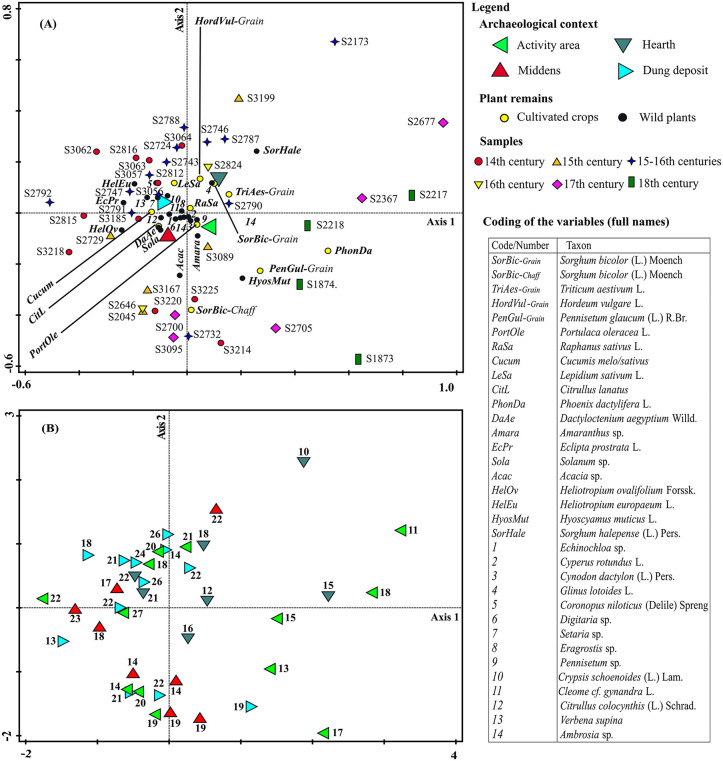
(A) Correspondence Analysis (CA) triplot showing the ordination of samples, plant species, and archaeological contexts, with three types of scores: sample positions, plant species, and archaeological contexts. **(B) Plant species diversity diagram illustrating changes in species diversity across samples within the ordination space,** with samples grouped by archaeological context to allow visual comparison of assemblages across context types. The first two CA axes explain a cumulative 30.18% of the total variation (Axis 1: 15.36%, Axis 2: 14.82%), while supplementary variables account for 7.1% of the total variation (Prepared by Mohammed Nasreldein).

The correspondence analysis (Fig 10) provides an exploratory ordination of samples and plant taxa, allowing a visual assessment of similarities in species composition across the assemblage. The first two axes explain 30.18% of the total variation (Axis 1: 15.36%, Axis 2: 14.82%), indicating that the observed patterns should be interpreted with caution. Archaeological contexts are included as supplementary groupings and are considered only as an aid to interpretation rather than as primary variables in the analysis. In addition, samples are grouped by archaeological context (see Fig 10B), allowing a visual comparison of plant assemblages across different context types within the site.

For instance, the CA triplot (Fig 10A) shows cultivated cereals like sorghum (*SorBic-Grain*), bread wheat (*TriAes-Grain*), and hulled barley (*HordVul-Grain*) clustering near the center of the plot, indicating that these taxa are widely distributed and common across most samples. Furthermore, the plot shows that they cluster closer to the hearth and activity area samples (e.g., S2842, S2790), indicating their widespread occurrence across samples, including those from domestic contexts. However, in the later periods, particularly the 17^th^ and 18^th^ centuries, bread wheat and hulled barley are much less frequent, while sorghum continues to dominate the assemblage, indicating a reduction in cereal diversity and possible changes in agricultural practices or food preferences. On the other hand, the wild and weedy plants – the small-seeded taxa – may be associated with contexts such as dung deposits and middens, although their presence could also reflect multiple pathways of deposition. The clustering of a large group of wild taxa in the centre suggests that wild plants are widely distributed across the assemblage, likely as fodder, potential food additives, weeds of the cultivated plants, or fuel-related remains.

The diachronic shift in plant consumption is reflected in the distinct clustering of samples by period. The 14^th^ century samples (red circles) tend to group toward the upper-left side of Axis 2, indicating a shared composition of plant remains characteristic of this earlier phase. In contrast, the 18^th^ century samples (green rectangles) are distinctly separated, clustering on the lower-right, along the positive side of Axis 1. This separation indicates differences in plant assemblages between periods. Samples S3214 and S3225 (14^th^ century) form exceptions by clustering with the 17^th^ and 18^th^ century samples. In the case of S3214, this is likely due to the presence of sorghum chaff remains (n = 88), a feature more typical of later assemblages. S3225, while lacking chaff remains, contains a discard pattern marked by purslane, watermelon, and radish seeds, and a high number of *Amaranthus* sp., further aligning it with the by-product-rich assemblages.

In general, samples from the 17^th^ and 18^th^ centuries show a shift toward fewer cultivated plants and greater emphasis on plants such as date palm (*PhonDa*), pearl millet (*PenGul-Grain*), wild sorghum (*SorHale*), and non-cultivated species like *Hyoscyamus muticus* L. (*HyosMut*) These later assemblages are also marked by the decline or disappearance of earlier garden crops, including radish (*RaSa*) and cress (*LeSa*) (see S2 Table). Additionally, 18^th^ century samples like S2217, S1873, and S2173 are positioned far from other clusters, showing a distinct composition, especially with taxa like *Phoenix dactylifera* (*PhonDa*) and *Sorghum halepense* (*SorHale*). This may reflect a narrowing of crop diversity or shifts in plant use toward the late Funj period.

**Fig 11:**
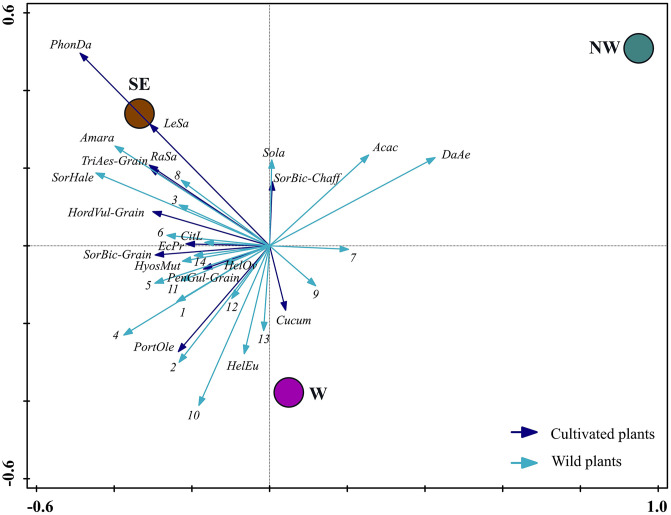
RDA (Redundancy Analysis) biplot showing the spatial distribution of plant taxa and archaeological units, based on constrained ordination by unit location. The analysis includes 18 samples from 12 archaeological units in the citadel area, spanning the West (W), Northwest (NW), and Southeast (SE), and comprises 34 identified plant taxa. Axis 1: Eigenvalue = 0.0720; cumulative explained fitted variation = 66.61%. Axis 2: Eigenvalue = 0.0361; cumulative explained fitted variation = 100.00%. Total inertia = 946.282. Location accounts for 10.8% of the fitted variation. For coding of explanatory variables, see Fig 10 and S3 File (Prepared by Mohammed Nasreldein).

The RDA (Redundancy Analysis) biplot (Fig 11) provides a constrained, exploratory assessment of spatial variation in plant assemblages based on sample location within the site: West (W), Northwest (NW), and Southeast (SE). The main edible cereals, including sorghum (*SorBic*-*Grain*), bread wheat (*TriAes*-*Grain*), hulled barley (*HordVul*-*Grain*), and pearl millet (*PenGul*-*Grain*) display a directional gradient rather than a uniform pattern. Bread wheat clusters closer to the SE, hulled barley lies between SE and W, sorghum occupies a slightly more central position, and pearl millet is oriented nearer to the West. This distribution indicates spatial variation in plant assemblages, although the underlying causes (e.g., differences in use, deposition, or activity areas) cannot be determined from this analysis alone.

In contrast, *SorBic-Chaff*, representing sorghum processing chaff remains, is positioned between the SE and NW, suggesting that more cereal processing activities were spatially concentrated in those areas. Other taxa are more evenly distributed between locations, occupying intermediate positions between directional vectors. Certain taxa, however, show clearer spatial associations. For instance, *PhonDa* (*Phoenix dactylifera* L.) and *LeSa* (*Lepidium sativum* L.) are strongly aligned with the SE, while taxa such as *DaAe* (*Dactyloctenium aegyptium* Willd.) and *Acac* (*Acacia* sp*.*) are more associated with the NW. The West (W) group displays a more moderate spread, with other plant taxa such as *HelEu* (*Heliotropium europaeum* L.), *PortOle* (*Portulaca oleracea* L.), and *Cucum* (*Cucumis melo/sativus*) distributed in its direction. Overall, the ordination indicates that some taxa are more closely associated with particular archaeological units, whereas others are present across multiple locations within the site.

These preliminary results indicate spatial patterning in the distribution of plant taxa across the site. For example, the positioning of sorghum chaff between the SE and NW areas may reflect differences in deposition or processing activities, although this cannot be determined with certainty from the present analysis alone. Variation in the distribution of cereals, such as bread wheat, hulled barley, sorghum, and pearl millet, may also point to differences in how these plants were used or deposited across the site. However, given the limited number of samples and the exploratory nature of the analysis, these patterns should be interpreted with caution, and further investigation is required to relate them to activity areas, unit function, or broader site organisation.

## Discussion

The archaeobotanical evidence from Old Dongola provides insight into diachronic changes in crop production, plant use, and dietary practices during a period of significant social and political transformation in Nubia. By comparing data from five centuries, we observe both continuity and change in subsistence strategies, including the persistence of staple cereals (sorghum, bread wheat, hulled barley, and pearl millet), the disappearance of certain crops (cotton, grape, anise, and cress), and the introduction of new ones (tobacco, eggplant, Egyptian henbane, and African peach) linked to trade networks and cultural exchange. The observed patterns of change across the occupation sequence at Old Dongola suggest shifts in subsistence strategy, particularly the increasing reliance on sorghum and pearl millet as staple cereal crops. This dominance of C4 plants such as sorghum and pearl millet, which are well adapted to dry conditions, aligns with broader regional trends in post-Medieval Nubia, where archaeobotanical evidence indicates intensified sorghum cultivation in the Middle Nile Region [38]. Archaeological evidence, including the frequent presence of *doka* plates (griddles) and the absence of bread ovens, further supports this interpretation and suggests changes in food preparation practices accompanying crop choice. This pattern may be considered in relation to both environmental conditions and socio-cultural factors, discussed further below.

The co-occurrence of weedy and economically important plants, including cultivated taxa alongside associated weed species, indicates that the assemblage primarily reflects anthropogenic activity (i.e., farming practices) rather than background environmental input, as such associations are typically linked to cultivation and crop-processing activities. Although not all taxa appear in every period, the recurring presence of both summer crops (e.g., sorghum, pearl millet, and cowpea) and winter crops (e.g., wheat, barley, and radish) throughout the sequence may point to the continued use of crops associated with different growing seasons at Old Dongola.

This interpretation is consistent with patterns documented elsewhere in Nubia, where *saqiya* irrigation supported year-round cultivation [25]. Fuller [81] interprets such patterns as evidence of agricultural intensification, potentially involving double-cropping, a concept aligned with Morrison’s [82] definition of agricultural intensification. Additionally, the crop diversity observed at Old Dongola reflected in the wide range of cultivated species (see S2 table) identified across different contexts and time periods aligns with what Morrison [82] defines as diversification: a complementary strategy involving staggered growing cycles, spatially varied plots, and a broad mix of species. While no formal diversity indices were applied, the taxonomic richness observed across assemblages supports this interpretation. Future work may benefit from applying formal diversity indices (e.g., Shannon or Simpson) to compare crop assemblages across periods more systematically.

The *saqiya*, a Persian-style cattle-powered waterwheel, appears to have been introduced into Nubia by the third century CE [1,5,24,25], although its widespread use and role in expanding cultivable land and diversifying cropping systems likely developed later. However, as Obłuski [3] points out, it was not merely the introduction but the consistent, large-scale use of *saqiya* that led to significant economic and social change.

Interestingly, at Qasr Ibrim, the widespread appearance of *saqiya* pots, locally known as *qawwadis* (sing. *qadus*) was closely associated with the introduction of new summer crops, highlighting how irrigation innovation and crop selection were mutually reinforcing [27]. At Old Dongola, recent research indicates a similar trajectory. Although no physical installations of the *saqiya* waterwheel have been discovered so far, *qawwadis* were frequently found in Funj period pottery assemblages [83]. According to Danys [83], these vessels comprised about 4.2% of the ceramic assemblage of the Funj period, and while their functions appear to have been multifunctional – including use as heating containers embedded in benches – they were primarily used in *saqiya* systems. Their presence, especially in comparison with earlier Makurian pottery types that lacked this multifunctional utility, suggests that the production of *qawwadis* intensified during the Funj period [84]. This aligns with the broader trend of increasing reliance on summer crops like sorghum and millet during this period, and is consistent with the interpretation that irrigation infrastructure may have played an important role in sustaining more arid-adapted, double-season cropping strategies.

The diversification in cropping systems including the presence of summer crops (e.g., sorghum and pearl millet) and winter crops (bread wheat and hulled barley), the variety of wild and other cultivated plants (e.g., purslane, watermelon, coriander, cress, safflower, and all the varieties of wild grasses), and the water-demanding or labour-intensive plant species (e.g., cotton, and cowpea) may reflect adaptive resilience to local environmental variability and resource constraints, as evidenced by patterns that may indicate flexible cropping practices. On the other hand, the increasing prominence of sorghum-based products such as *kisra* and *asida* over wheat bread aligns with historical accounts of shifting agricultural practices and crop preferences during the Funj period [38,85,86]. This period saw a growing emphasis on indigenous African crops, including sorghum, millet, and pulses, which were likely both more accessible and better suited to prevailing environmental conditions than wheat and barley.

This transition may reflect both environmental and cultural factors. In environmental terms, proxy climate records suggest a gradual shift toward increasingly arid conditions in the Middle Nile Valley after the first millennium BC [87,88], a regional trend that may have intensified during the post-Meroitic (350–500 CE) and later periods. Such conditions would have favoured crops such as sorghum and millet, which are better adapted to lower water availability. Recent isotopic studies from human and faunal remains also indicate increased reliance on C4 plants during the post-Meroitic and medieval periods [89–91].

These changes in dietary and crop preferences likely coincided with shifts in irrigation practices, as communities may have responded to increasingly arid conditions in the Middle Nile Valley, including reduced rainfall and less predictable flooding regimes, by intensifying the use of existing technologies, such as the *saqiya*, to support summer cultivation [25]. In this context, the continued rise of sorghum at Old Dongola likely reflects environmental suitability in the first instance. It may also be related to changing cultural practices, such as a gradual movement away from older Near Eastern influences toward a more localized Sudanese dietary tradition rooted in African staple crops [38,85,86]. This is potentially linked to broader cultural and political developments during the Funj period, which Spaulding [92] has characterized as a Nubian renaissance within an Islamic framework. It may further reflect changing socio-economic dynamics, as locally grown, drought-tolerant crops like sorghum became increasingly central to subsistence and cultural identity in the region.

This adaptive cropping strategy also finds parallels in archaeobotanical evidence from elsewhere in Nubia. At Qasr Ibrim, new crops, particularly summer-grown plants such as sorghum and cotton, appear in the record by the first millennium CE [27], indicating an increasing emphasis on summer cultivation. These developments have often been discussed in relation to Watson’s concept of an “Agricultural Revolution” in the Islamic world, which highlights the wider adoption of tropical-origin crops and the expansion of summer growing seasons [93] and may provide a broader comparative framework for interpreting similar patterns at Old Dongola. However, the Nubian evidence suggests that such agricultural adjustments were already underway prior to Islamic political control, pointing to locally driven processes of experimentation and adaptation rather than changes directly tied to religious or political transitions. While the available data remains limited to a small number of well-studied sites, including Qasr Ibrim and to some extent Old Dongola, it nevertheless highlights the capacity of Nubian agricultural systems to respond flexibly to environmental and social conditions. In this sense, the cropping patterns observed at Old Dongola, including the dominance of sorghum, the gradual disappearance of bread wheat and hulled barley, and the incorporation of summer crops such as watermelon and safflower, may reflect Nubia’s role as an active zone of agricultural experimentation and adaptation that participated in wider regional developments.

Bread wheat, despite its importance, may have been used in Nubia more domestically than commercially; sources indicate that wheat was often exchanged for other crops, for instance pearl millet [7]. Oral histories from the Old Dongola region indicate that wheat was cultivated only in small quantities for household use rather than as a market crop, and this pattern aligns with its limited presence in the archaeobotanical assemblages, including those from the 16^th^ century onward (see S2 Table). Given that wheat is generally less well adapted to arid conditions than crops such as sorghum or millet, this reduction in cultivation may be related to broader environmental pressures, particularly increasing aridity in the Middle Nile Valley. However, as irrigation systems such as the *saqiya* were available, this shift was likely not determined by environmental factors alone but may also reflect cultural and economic preferences in crop selection and use [38,53,94]. This interpretation is consistent with previously discussed paleoenvironmental studies indicating increasing dryness in the region after the first millennium BC [87,88,90], suggesting that the declining presence of bread wheat and hulled barley reflects a combination of environmental pressures, irrigation changes, and shifting agricultural priorities.

During periods of agricultural scarcity, Nubian communities likely relied more heavily on existing village-based exchange systems (barter) to supplement their needs, exchanging goods such as dates, wheat, and pulses, while the circulation of larger quantities was primarily managed by elite or royal actors operating through major trading centres [7]. In this context, wheat functioned less as a staple and more as a valuable commodity within local commerce, traded for more drought-tolerant crops like pearl millet. This shift may also reflect a gradual cultural reorientation toward African staple crops, as sorghum and millet held deeper roots in local dietary traditions and increasingly came to signify a distinctly ancient Sudanese/Nubian food identity during the Funj period [38].

Pulses, while present, are generally underrepresented in the assemblage. Cowpea (*Vigna unguiculata*) appears sporadically, and this scarcity is likely influenced by preservation biases, although context-related factors cannot be entirely excluded. Given the systematic sampling of contexts within the excavated areas (see S1 Table), this pattern is unlikely to reflect recovery bias. Similarly, safflower (*Carthamus tinctorius* L.), a crop with multiple uses such as oil and dye production, appears in notable quantities in 16^th^ century contexts but is absent from later assemblages; this contrast may instead be influenced by the limited spatial coverage of excavation across the site.

Cotton (*Gossypium* sp.) is recorded only in two contexts from the 14^th^ century, raising questions about its cultivation during later periods. The absence of cotton in the Funj period contexts may reflect sampling bias but could also indicate a reconfiguration of production priorities. Oral histories and historical records suggest cotton remained important locally, so future studies will be needed to resolve this question. Historical sources support the presence of cotton in medieval Nubia [2]. According to Ibn Salim al-Uswānī, in the 10^th^ century, Nubians of Dongola only maintained small plots of cotton, which were likely used for producing coarse clothing [2,30].

The presence of both imported and locally produced cotton textiles at Old Dongola during the Funj period, as indicated by textile finds and associated analysis [95,96], suggests active textile production and exchange, even though direct archaeobotanical evidence for cotton cultivation is absent. Rather than signalling a cessation of cotton cultivation after the 14^th^ century, this absence is more plausibly explained by limitations in archaeobotanical recovery and by variability in household-level production and discard practices. Comparative evidence from Qasr Ibrim [97] further suggests that cotton was well adapted to regional environmental conditions, supporting the likelihood that cotton continued to circulate within local and regional economic networks, even when it is not visible in the archaeobotanical record.

Within this context, cotton at Old Dongola may have been cultivated locally without leaving a clear archaeobotanical signature or alternatively acquired as raw fibre or finished textiles through trade. Its archaeological invisibility in Funj-period contexts may reflect preservation constraints or changes in processing and distribution rather than agricultural abandonment. Although repeated attempts at DNA extraction from the Old Dongola cotton sample were unsuccessful, likely due to low DNA concentration or inhibitory residues, archaeogenomic research from Qasr Ibrim has identified cotton remains as *Gossypium herbaceum*, an African species probably domesticated locally [97]. Together, these findings highlight the need to interpret the absence of cotton remains at Old Dongola with caution and within a broader regional framework.

Other crops display more consistent patterns that may reflect domestic use and shifting cultivation, based on their distribution across the sampled contexts. Some plants, like coriander (*Coriandrum sativum* L.) and black nightshade (*Solanum nigrum* L.), are consistently present in earlier periods (14^th^–15^th^ centuries) but disappear in later assemblages, suggesting possible changes in cultivation conditions as well as shifts in dietary preferences and culinary practices over time. The rare occurrence of condiment plants in general, along with safflower and possibly cotton, may indicate changing agricultural priorities, evolving food habits, or a gradual loss of certain cultivation knowledge. Determining whether these patterns reflect cultural choices, environmental constraints, or changes in the transmission of agricultural knowledge requires closer comparison with other Nubian and Egyptian sites. For instance, the work at Qasr Ibrim [27,98], Sai island [28], Nauri [41], and Soba East [40,42] has revealed similar reductions in winter crops during the medieval and post-medieval periods, although direct botanical comparisons remain limited due to sample size and preservation differences.

The Kingdom of Dongola and the rise of Islam in the region coincide with the appearance of new cultivated plants. For instance, tobacco (*Nicotiana tabacum* L.) appears from the second half of the 16^th^ century onward and increases in the 18^th^ century [26]. Its early presence suggests adoption through trade networks involving merchants and migrants [10,26,48,99]. Additionally, Egyptian henbane (*Hyoscyamus muticus* L.) also first appears in this period and increases in frequency over time, while eggplant (*Solanum melongena* L.) is briefly present before disappearing until the 18^th^ century. The arrival and intermittent use of these plants points to expanding trade routes and cultural exchange during this transformative period in Nubian history. These introduced plants may not only reflect new economic networks but also shifting symbolic or cultural practices. In particular, henbane and tobacco both carry psychoactive or ritual significance in other Islamic and African cultural contexts [26,100–103]. Their use at Old Dongola may relate to changing social, healing, or ceremonial behaviours, though further contextual evidence is needed.

Noteworthy among the assemblage is the identification of African peach (*Nauclea latifolia*), and false daisy (*Eclipta prostrata* L*.*), two species not previously reported in archaeobotanical studies from the region [104]. While some of these may reflect localized or short-term use, their presence broadens our understanding of plant diversity and cultural knowledge in medieval and post-medieval Nubia. These first-time identifications suggest a potentially underexplored range of plant use that future work – including broader sampling, and ethnographic engagement – could elaborate further.

### Grapes, viticulture, and the legacy of wine in Nubia

The assemblage reflects the use of a variety of fruits, including fig, grape, date, and melon/cucumber, though some of these disappear in later periods. The restricted appearance of grapes, only in the 14^th^ century assemblage, is noteworthy, although the limited number of remains makes interpretation cautious. Only ten desiccated and fragmented grape seeds were recovered, and their limited preservation makes species-level identification difficult. However, the fragmented nature of these remains may be consistent with grape processing, including as possible by-products of wine production. As noted by El Dorry [105], pressed grape by-products can be indicative of winemaking. While viticulture was likely rare in Nubia, previous archaeobotanical finds and inscriptions from sites such as Kawa [106] and later Roman-style wine presses in Lower Nubia [1,33,107] suggest localized efforts to cultivate grapes through adopting *saqiya* irrigation techniques. The presence of wine presses at sites such as Meroe, Meinarti, and Wadi el-Arab during the 3^rd^–4^th^ centuries CE is often interpreted as a Meroitic attempt – perhaps influenced by Greek or Egyptian techniques – to establish viticulture, although the eventual abandonment of these facilities suggests that climate conditions posed significant challenges [1,33,42,108].

A comparative case comes from Soba East, where grape pips were discovered in association with church structures, potentially part of a monastic unit [42]. Though the climate in the Middle Nile Region is too hot and dry for grape cultivation today, the finds suggest either small-scale, irrigation-supported viticulture or the import of dried grapes or raisins. According to van der Veen and Lawrence [42], the association with monastic buildings echoes Mediterranean patterns in which grape cultivation was often linked to ecclesiastical institutions.

At Old Dongola, according to Godlewski [20] the archaeological evidence highlights the significance of wine consumption and possibly localized production during the Makurian period. Hundreds of amphora fragments – many imported from Egypt and Palestine – were discovered in elite palace contexts, alongside stamped mud stoppers and lime mortar caps, possibly reflecting long-distance trade and controlled distribution [47,109]. Importantly, Godlewski [20] highlighted that the sudden increase in the production of locally-made amphorae at Dongola during the 7^th^ century may indicate the adaptation of grapevines to local conditions and the emergence of local winemaking, likely supported by the fertility of the Letti basin and the use of *saqiya* irrigation.

The continued importance of wine into the Islamic period is reflected in the *baqt* agreement, a long-standing political and economic treaty between Christian Makuria and Muslim Egypt, established after the Arab conquest of Egypt in 641 CE which regulated trade, tribute, and peaceful coexistence across the two regions [1,48,110]. Wine was among several luxury imports listed in historical sources, alongside wheat, olive oil, and fine textiles, in exchange for goods such as ivory, exotic animals, and slaves [110]. This broader historical and archaeological context reinforces the importance of viticulture and wine at Old Dongola during the Christian Makurian period. The absence of grape remains in later Funj period contexts may most plausibly be linked to the broader cultural and religious transformations associated with the transition from the Christian Makurian period to the Islamic period, where the decline of viticulture and grape consumption would be consistent with Islamic dietary practices, although such interpretations remain tentative given the limited archaeobotanical evidence. Changes in agricultural practices, trade networks, or consumption patterns may have also contributed to this absence.

While wine functioned as a marker of elite status and ritual practice during the Christian Makurian period, its disappearance from later contexts may reflect not only shifting political and religious affiliations but also the decline of Christian elite institutions that had linked Nubia to the Mediterranean world. In contrast to wine, fermented and unfermented beverages made from sorghum (*marissa, beer,* and *bouza*) or dates (*aragi* and *dakkai*) have long constituted part of daily Nubian consumption and social rituals persisting into recent memory [2,35,86]. This pattern may be viewed within a broader framework of changing consumption practices, in which Christian-associated products such as wine appear less visible in the archaeobotanical record, while locally rooted beverages remain prominent.

## Conclusion

This study presents the first systematic archaeobotanical analysis of Old Dongola and provides new evidence for plant use, agriculture, and food practices in the medieval and post-medieval Middle Nile Valley. By focusing on a region where archaeobotanical data remain scarce, it contributes a much-needed empirical foundation for discussions of agricultural and dietary change in precolonial Sudan.

The results from Old Dongola indicate that agricultural and food practices between the 14^th^ and 18^th^ centuries CE were characterized by both continuity and selective change. Rather than sudden transformations, the archaeobotanical assemblage reflects gradual adjustments in crop choices, cultivation strategies, and consumption practices, shaped by environmental conditions, cultural traditions, and shifting social structures. The prominence of locally adapted crops and beverages highlights the resilience of Nubian foodways and the capacity of communities to negotiate broader historical transitions through flexible subsistence strategies.

At the same time, this study highlights important limitations in the available dataset. The absence or decline of certain crops, including cotton, cowpea, and safflower, should not be interpreted as definitive evidence for their abandonment, but rather as a reflection of restricted sampling, household-level variability, and the limited spatial extent of excavation at Old Dongola. Archaeobotanical visibility is inevitably shaped by recovery methods, preservation conditions, and the specific areas investigated, and much of the settlement remains unexplored.

As a result, Old Dongola should be understood as a well-documented case study rather than a comprehensive proxy for Nubia as a whole. Future research combining expanded archaeobotanical sampling, textile and artifact studies, and biomolecular approaches across multiple sites will be essential for refining regional interpretations of agricultural production, exchange networks, and dietary practices. Nonetheless, the evidence presented here demonstrates the value of archaeobotany for reconstructing long-term patterns of adaptation and continuity in the Middle Nile Valley and provides a firm basis for comparative work in the region.

## Supporting information

S1 Table Summary of sediment samples analyzed in this study, including contextual and preservation details. (DOCX)

S2 Table Chronological summary of plant remains recovered from Old Dongola, showing quantitative changes and ubiquity trends across the historical periods. (DOCX)

S3 Table Coding of the variables in the correspondence analysis. (DOCX)

S4 File Correspondence Analysis (CA) data. (XLSX)

S5 File Spatial Analysis Data. (XLSX)

S6 File Inclusivity in global research. (DOCX)
