# Supplementary material for: Continuity or change in the transition to Islam? A diachronic assessment of agricultural production at Old Dongola, Northern Sudan (14th–18th centuries CE)
Source: PLoS One. 2026 Jul 9;21(7):e0353303. doi: 10.1371/journal.pone.0353303 (PMC13349140; doi:10.1371/journal.pone.0353303)
Supplement: S1 Table — (DOCX) [file pone.0353303.s001.docx]

S1. Table. Summary of sediment samples analysed in this study, including contextual and preservation details.

| **Period** | **Excavation Unit** | **Context No.** | **Context Description** | **Sample No.** | **Sediment Volume (L)** | **Field Assessment** | **Preservation of seeds** | |
| --- | --- | --- | --- | --- | --- | --- | --- | --- |
|  |  |  |  |  |  |  | **Desiccated** | **Charred** |
| 14^th^ C. | U244 | 1580 | Dung deposits; and organic matters | 2812 | 10 | Flotation | 692 | - |
| 14^th^ C. | U244 | 1580 | Fine sands; ashes; and organic matters | 2815 | 10 | Flotation | 767 | 2 |
| 14^th^ C. | U244 | 1580 | Dung deposits; and organic matters | 2816 | 12 | Flotation | 1045 | - |
| 14^th^ C. | U257 | 1622 | Fine sands; rubbles and organic matters | 3185 | 13 | Flotation | 774 | 1 |
| 14^th^ C. | U244 | 1669 | Dung deposits; and organic matters | 3062 | 15 | Flotation | 491 | - |
| 14^th^ C. | U244 | 1669 | Dung deposits; and organic matters | 3063 | 12 | Flotation | 1280 | 1 |
| 14^th^ C. | U244 | 1669 | Dung deposits; and organic matters | 3064 | 15 | Flotation | 247 | - |
| 14^th^ C. | U259 | 1692 | Ashes; rubbles; dung deposits with organic matters | 3214 | 15 | Flotation | 866 | 10 |
| 14^th^ C. | U244 | 1693 | Ashes; rubbles; dung deposits with organic matters | 3213 | 15 | Flotation | 76 | 204 |
| 14^th^ C. | U244 | 1694 | Fine sands; dung deposits; and organic matters | 3220 | 15 | Flotation | 428 | - |
| 14^th^ C. | U244 | 1694 | Fine sands; dung deposits; and organic matters | 3218 | 15 | Flotation | 378 | - |
| 14^th^ C. | U275 | 1699 | Ashy soil; rubbles; charcoal, and organic remains | 3225 | 15 | Flotation | 173 | 70 |
| 15^th^ C. | U174 | 1136 | Dung deposits mixed with organic matters | 2045 | 15 | Flotation | 430 | - |
| 15^th^ C. | U235 | 1606 | Ashy-Sandy soil; dung deposits; and organic matters | 3167 | 12 | Flotation | 103 | 61 |
| 15^th^ C. | U256 | 1684 | Ashes; charcoal; and burnt matters | 3077 | 15 | Flotation | - | 483 |
| 15^th^ C. | U241 | 1686 | Ashes; and burnt organic matters | 3089 | 13 | Flotation | 699 | 13 |
| 15^th^ C. | U210 | F2172 | clayey soil; rubbles; and debris | 2729 | 7 | Flotation | 732 | - |
| 15^th^ C. | U278 | 1811(FN1133) | Filling of a cooking pot, seeds and organic matters | 3199 | 5 | Dry sieving | 2763 | - |
| 15^th^-16^th^ C. | U214 | 1180 | Dung deposits; ashes; and chaff remains | 2724 | 8 | Flotation | 1382 | 1 |
| 15^th^-16^th^ C. | U214 | 1185 | Dung deposits; ashes; and chaff remains | 2743 | 10 | Flotation | 605 | - |
| 15^th^-16^th^ C. | U214 | 1185 | Dung deposits; ashes; and chaff remains | 2746 | 15 | Flotation | 1272 | - |
| 15^th^-16^th^ C. | U214 | 1185 | Fine sands mixed with organic matters | 2747 | 11 | Flotation | 788 | 16 |
| 15^th^-16^th^ C. | U214 | 1185 | Hearth; ashes; and organic matters | 2787 | 15 | Flotation | 768 | 95 |
| 15^th^-16^th^ C. | U207 | 1186 | Ashy-Sandy soil, dung deposits; and organic matters | 2732 | 15 | Flotation | 742 | - |
| 15^th^-16^th^ C. | U100 | 1257 | Ashes and charcoal; an occupational layer in a kitchen space | 2173 | 10 | Flotation | - | 109 |
| 15^th^-16^th^ C. | U214 | 1553 | Fine sands; dung deposits; and organic matters | 2792 | 13 | Flotation | 877 | - |
| 15^th^-16^th^ C. | U214 | 1554 | Dung deposits; and organic matters | 2788 | 15 | Dry sieving & Flotation | 1762 | - |
| 15^th^-16^th^ C. | U214 | 1556 | Ashes and burnt organic matters | 2790 | 12 | Flotation | - | 127 |
| 15^th^-16^th^ C. | U214 | 1557 | Dung deposit; organic matters; and plant remains | 2791 | 13 | Flotation | 1566 | - |
| 15^th^-16^th^ C. | U214 | 1666 | Ashes; charcoal; and burnt matters (dump) | 3056 | 15 | Flotation | 381 | 3 |
| 15^th^-16^th^ C. | U214 | 1666 | Ashes; charcoal; and burnt matters (dump) | 3057 | 12 | Flotation | 703 | 53 |
| 15^th^-16^th^ C. | U214 | 1674 | Ashes; charcoal; and burnt organic matters | 3066 | 9 | Flotation | 96 | 84 |
| 16^th^ C. | U214 | 1664 | Dung deposits; and organic matters | 2824 | 15 | Flotation | 906 | - |
| 16^th^ C. | U212 | 1473 | Fine sands; dung deposits; and organic matters | 2646 | 10 | Flotation | 641 | - |
| 17^th^ C. | U115 | 1260 | Ashes; charcoal; fill of a fire pit in kitchen space | 2175 | 8 | Flotation | - | 10 |
| 17^th^ C. | U190 | 1392 | Ashes and charcoal; an occupational hearth layer in room U190 | 2367 | 10 | Flotation | 254 | 68 |
| 17^th^ C. | U223 | F2130 | Fill of a depression in the floor of room U223, occupied by basket FN930; organic matters and plant remains | 2677 | 1 | Flotation | 170 | - |
| 17^th^ C. | U230 | 1711 | Dung deposits; and organic matters | 2700 | 12 | Flotation | 503 | 14 |
| 17^th^ C. | U251 | 1721 | Dung deposits; and organic matters | 2705 | 8 | Flotation | 397 | 1 |
| 17^th^ C. | U251 | 1728 | Rubbles mixed with organic matters | 3095 | 8 | Flotation | 529 | - |
| 18^th^ C. | U112 | 969 | Rubbles mixed with organic matters | 1873 | 11 | Flotation | 267 | 1 |
| 18^th^ C. | U112 | 969 | Rubbles mixed with organic matters | 1874 | 10 | Flotation | 460 | 1 |
| 18^th^ C. | U112 | 969 | Ashes and charcoal | 2217 | 10 | Flotation | 343 | 1 |
| 18^th^ C. | U112 | 969 | Ashes and charcoal | 2218 | 10 | Flotation | 376 | 2 |
