# Supplementary material for: Continuity or change in the transition to Islam? A diachronic assessment of agricultural production at Old Dongola, Northern Sudan (14th–18th centuries CE)
Source: PLoS One. 2026 Jul 9;21(7):e0353303. doi: 10.1371/journal.pone.0353303 (PMC13349140; doi:10.1371/journal.pone.0353303)
Supplement: S2 Table — (DOCX) [file pone.0353303.s002.docx]

Table 1. Chronological summary of plant remains recovered from Old Dongola, showing quantitative changes and ubiquity trends across the historical periods.

| **Period** | **14th C.** | | **15th C.** | | **15-16th C.** | | **16th C.** | | **17th C.** | | | **18th C.** | |  |  |  |  |  |
| --- | --- | --- | --- | --- | --- | --- | --- | --- | --- | --- | --- | --- | --- | --- | --- | --- | --- | --- |
| **Number of samples** | 12 | | 6 | | 14 | | 2 | | 6 | | | 4 | |  |  |  |  |  |
| **Sediment Volume (L)** | 162 | | 67 | | 173 | | 25 | | 47 | | | 41 | |  |  |  |  |  |
| **Taxa** |  | |  | |  | |  | |  | | |  | |  |  |  |  |  |
| **Cereals** | **Prop. %** | **Ub. %** | **Prop. %** | **Ub. %** | **Prop. %** | **Ub. %** | **Prop. %** | **Ub. %** | **Prop. %** | **Ub. %** | | **Prop. %** | **Ub. %** | **Total** | **Overall Ubiquity (%)** | **English Name** | **Vernacular Name (Arabic/Nubian)** |  |
| *Sorghum bicolor* (L.) Moench | 8.96% | 91.67% | 18.41% | 66.67% | 10.0% | 100.0% | 19.39% | 100.0% | 11.31% | 66.67% | | 7.79% | 75.00% | 3412 | 86% | Sorghum | durra / marii |  |
| *Triticum aestivum* L. | 0.19% | 33.33% | 10.43% | 50.0% | 0.25% | 28.57% | 0.0% | 0.0% | 0.21% | 16.67% | | 0.55% | 50.0% | 605 | 32% | Bread wheat | gamih / ellee |  |
| *Hordeum vulgare* L. | 2.21% | 66.67% | 7.55% | 100.0% | 2.14% | 78.57% | 1.23% | 50.0% | 2.93% | 66.67% | | 0.55% | 75.00% | 891 | 75% | Hulled barley | sha’eer / seringi |  |
| *Pennisetum glaucum* (L.) R.Br. | 0.19% | 16.67% | 0.87% | 50.0% | 0.0% | 0.0% | 0.97% | 50.0% | 0.15% | 33.33% | | 1.52% | 75.00% | 100 | 25% | Pearl millet | dukhn / foto |  |
| **Pulses** |  |  |  |  |  |  |  |  |  |  | |  |  |  |  |  |  |  |
| *Lupinus albus* L. | 0.15% | 33.33% | 0.04% | 16.67% | 0.03% | 7.14% | 0.0% | 0.0% | 0.10% | 33.33% | | 0.0% | 0.0% | 25 | 16% | Termis | turmus |  |
| *Vigna unguiculata* (L.) Walp. | 0.0% | 0.0% | 0.06% | 16.67% | 0.01% | 7.14% | 0.0% | 0.0% | 0.0% | 0.0% | | 0.0% | 0.0% | 4 | 5% | Cowpea | lubia helu / digintee |  |
| *Lathyrus sativus* L. | 0.0% | 0.0% | 0.0% | 0.0% | 0.0% | 0.0% | 0.0% | 0.0% | 0.0% | 0.0% | | 0.07% | 25.00% | 1 | 2% | Grass pea | bisilla / guuru |  |
| *Astragulus* sp. | 0.0% | 0.0% | 0.09% | 16.67% | 0.0% | 0.0% | 0.0% | 0.0% | 0.05% | 16.67% | | 0.0% | 0.0% | 6 | 5% | Milkvetch | qatād |  |
| **Condiments, herbs, and spices** |  |  |  |  |  |  |  |  |  |  | |  |  |  |  |  |  |  |
| *Mutarda nigra* (L.) Bernh*.* | 0.16% | 16.67% | 0.49% | 16.67% | 0.05% | 21.43% | 0.0% | 0.0% | 1.13% | 16.67% | | 0.0% | 0.0% | 66 | 16% | Black mustard | khardal |  |
| *Coriandrum sativum* L. | 0.03% | 8.33% | 0.06% | 33.33% | 0.02% | 7.14% | 0.0% | 0.0% | 0.0% | 0.0% | | 0.0% | 0.0% | 7 | 9% | khardal | khardal |  |
| *Lepidium sativum* L. | 0.15% | 33.33% | 0.06% | 50.0% | 0.10% | 28.57% | 0.26% | 1.00% | 0.0% | 0.0% | | 0.0% | 0.0% | 29 | 23% | Cress | el-rashaad |  |
| *Pimpinella anisum* L. | 0.04% | 8.33% | 0.0% | 0.0% | 0.0% | 0.0% | 0.0% | 0.0% | 0.0% | 0.0% | | 0.0% | 0.0% | 3 | 2% | Anise | harjal |  |
| *Ceratonia siliqua* L. | 0.0% | 0.0% | 0.04% | 16.67% | 0.01% | 7.14% | 0.0% | 0.0% | 0.0% | 0.0% | | 0.0% | 0.0% | 3 | 5% | Carob | el-kharrub |  |
| *Senna* sp*.* | 0.0% | 0.0% | 0.0% | 0.0% | 0.0% | 0.0% | 0.13% | 50.0% | 0.0% | 0.0% | | 0.0% | 0.0% | 2 | 2% | Senna | senna maka |  |
| *Acacia* sp. | 0.24% | 41.67% | 0.21% | 33.33% | 0.09% | 28.57% | 0.0% | 0.0% | 0.26% | 33.33% | | 0.14% | 25.00% | 46 | 32% | Acacia | sonot; talih; hashab |  |
| *Ambrosia* sp. | 0.43% | 41.67% | 0.04% | 16.67% | 0.04% | 21.43% | 0.39% | 100.0% | 0.15% | 33.33% | | 0.21% | 25.00% | 50 | 32% | Ragweed | damsisa |  |
| **Fruits** |  |  |  |  |  |  |  |  |  |  | |  |  |  |  |  |  |  |
| *Citrullus lanatus* (Thunb.) Matsum. & Nakai | 0.87% | 50.0% | 0.0% | 0.0% | 0.22% | 42.86% | 0.78% | 50.0% | 0.26% | 33.33% | | 0.0% | 0.0% | 107 | 34% | Watermelon | battikh |  |
| *Ficus* sp. | 0.04% | 16.67% | 0.0% | 0.0% | 0.01% | 7.14% | 0.0% | 0.0% | 0.0% | 0.0% | | 0.34% | 50.0% | 9 | 11% | Fig | teen |  |
| *Phoenix dactylifera* L. | 0.08% | 16.67% | 0.0% | 0.0% | 0.01% | 7.14% | 0.0% | 0.0% | 1.49% | 66.67% | | 4.14% | 100.0% | 96 | 25% | Palm date | nakheel/tamur / fanti |  |
| *Vitis* sp*.* | 0.13% | 25.00% | 0.0% | 0.0% | 0.0% | 0.0% | 0.0% | 0.0% | 0.0% | 0.0% | | 0.0% | 0.0% | 10 | 7% | Grape | einab |  |
| *Cucumis melo/sativus* | 1.93% | 83.33% | 0.57% | 83.33% | 0.80% | 64.29% | 0.90% | 100.0% | 0.72% | 50.0% | | 0.0% | 0.0% | 294 | 66% | Cucumber/Melon | agour/shamam |  |
| *Nauclea latifolia* Sm*.* | 0.0% | 0.0% | 0.0% | 0.0% | 0.01% | 7.14% | 0.0% | 0.0% | 0.10% | 33.33% | | 0.21% | 75.00% | 6 | 11% | African peach | el-khawkh el-ifriqi |  |
| *Ziziphus* sp. | 0.03% | 16.67% | 0.0% | 0.0% | 0.0% | 0.0% | 0.0% | 0.0% | 0.0% | 0.0% | | 0.0% | 0.0% | 2 | 5% | Jujube | sidr / nabagka |  |
| *Capparis* cf. *decidua* | 0.0% | 0.0% | 0.0% | 0.0% | 0.02% | 7.14% | 0.0% | 0.0% | 0.0% | 0.0% | | 0.62% | 50.0% | 11 | 7% | Karir | tundub / **garae** |  |
| **Fibre/Oil crops** |  |  |  |  |  |  |  |  |  |  | |  |  |  |  |  |  |  |
| *Gossypium* sp. | 0.24% | 16.67% | 0.0% | 0.0% | 0.0% | 0.0% | 0.0% | 0.0% | 0.0% | 0.0% | | 0.0% | 0.0% | 18 | 5% | Cotton | Gottun / koshmag |  |
| *Carthamus tinctorius* L. | 0.61% | 41.67% | 0.26% | 16.67% | 0.0% | 0.0% | 8.73% | 50.0% | 0.0% | 0.0% | | 0.0% | 0.0% | 195 | 16% | Safflower | el- ‘usfur / kusheeg |  |
| *Raphanus sativus* L. | 0.57% | 58.33% | 0.55% | 66.67% | 0.71% | 57.14% | 1.16% | 100.0% | 0.41% | 33.33% | | 0.62% | 50.0% | 188 | 57% | Radish | figel |  |
| **Wild plants** |  |  |  |  |  |  |  |  |  |  | |  |  |  |  |  |  |  |
| *Echinochloa* sp. | 20.81% | 100.0% | 14.40% | 100.0% | 24.29% | 85.71% | 14.67% | 100.0% | 22.40% | 83.33% | | 2.28% | 75.00% | 5772 | 91% | Jungle Rice | defra |  |
| *Cyperus rotundus* L. | 24.80% | 100.0% | 15.71% | 100.0% | 21.54% | 92.86% | 11.25% | 100.0% | 18.81% | 100.0% | | 14.00% | 100.0% | 5875 | 98% | Nut grass | seida |  |
| *Cynodon dactylon* (L.) Pers. | 5.97% | 100.0% | 4.52% | 66.67% | 6.65% | 78.57% | 14.54% | 100.0% | 4.37% | 66.67% | | 14.83% | 100.0% | 1966 | 84% | Bermuda grass | nagiila / bunddi |  |
| *Glinus lotoides* L*.* | 8.72% | 58.33% | 9.78% | 33.33% | 5.21% | 71.43% | 10.08% | 50.0% | 4.62% | 50.0% | | 12.97% | 75.00% | 2197 | 59% | _ | rabaat el-Bahr / rabaat |  |
| *Coronopus niloticus* (Delile) Spreng. | 2.65% | 58.33% | 0.72% | 50.0% | 2.13% | 64.29% | 2.46% | 100.0% | 0.41% | 33.33% | | 0.0% | 0.0% | 524 | 52% | Swinecress | _ |  |
| *Digitaria* sp. | 0.43% | 58.33% | 0.42% | 83.33% | 0.94% | 78.57% | 0.06% | 50.0% | 2.11% | 66.67% | | 0.07% | 25.00% | 204 | 66% | Crabgrass | sha’ar el-banaat |  |
| *Setaria* sp. Ttype 1 | 0.73% | 58.33% | 0.13% | 16.67% | 1.16% | 78.57% | 0.19% | 50.0% | 0.36% | 16.67% | | 0.0% | 0.0% | 203 | 48% | Foxtail grass | losseig |  |
| *Setaria* sp. Type 2 | 0.03% | 8.33% | 1.08% | 16.67% | 0.42% | 35.71% | 0.0% | 0.0% | 0.0% | 0.0% | | 0.0% | 0.0% | 107 | 16% | Foxtail grass | losseig |  |
| *Setaria* sp. Type 3 | 0.29% | 8.33% | 0.0% | 0.0% | 0.36% | 21.43% | 0.0% | 0.0% | 0.0% | 0.0% | | 0.0% | 0.0% | 63 | 9% | Foxtail grass | losseig |  |
| *Eragrostis* sp. Type 1 | 1.69% | 66.67% | 3.07% | 50.0% | 3.04% | 85.71% | 2.13% | 100.0% | 1.70% | 66.67% | | 1.31% | 100.0% | 719 | 75% | Lovegrass | halagoya / toshe |  |
| *Eragrostis* sp. Type 2 | 0.0% | 0.0% | 0.0% | 0.0% | 0.0% | 0.0% | 5.04% | 50.0% | 0.62% | 16.67% | | 0.0% | 0.0% | 90 | 5% | Lovegrass | halagoya / toshe |  |
| *Pennisetum* sp. | 1.52% | 50.0% | 0.79% | 50.0% | 1.38% | 64.29% | 0.26% | 50.0% | 0.41% | 16.67% | | 1.10% | 50.0% | 340 | 50% | Elephant grass | _ |  |
| *Dactyloctenium aegyptium* Willd. | 0.0% | 0.0% | 0.23% | 33.33% | 0.13% | 42.86% | 0.0% | 0.0% | 0.15% | 16.67% | | 0.07% | 25.00% | 31 | 23% | Crowfoot grass | um assabi / koreib |  |
| *Crypsis schoenoides* (L.) Lam*.* | 0.96% | 58.33% | 1.14% | 66.67% | 1.28% | 64.29% | 0.71% | 100.0% | 0.51% | 50.0% | | 0.07% | 25.00% | 299 | 59% | Swamp pricklegrass | | |
| *Amaranthus* sp*.* | 1.33% | 8.00% | 0.30% | 66.67% | 0.71% | 78.57% | 0.45% | 50.0% | 8.17% | 83.33% | | 2.97% | 100.0% | 406 | 75% | Pigweed | lisan el-teir | |
| *Solanum* sp. | 0.47% | 83.33% | 0.06% | 33.33% | 0.10% | 14.29% | 0.13% | 50.0% | 0.21% | 33.33% | | 0.55% | 100.0% | 63 | 48% | Nightshade | gubbein | |
| *Solanum nigrum* L. | 0.01% | 8.33% | 0.09% | 33.33% | 0.10% | 21.43% | 0.0% | 0.0% | 0.0% | 0.0% | | 0.0% | 0.0% | 17 | 14% | Black nightshade | einab el-diib / gubbein | |
| *Verbena supina* | 0.49% | 58.33% | 0.26% | 50.0% | 0.61% | 50.0% | 0.19% | 50.0% | 0.0% | 0.0% | | 0.14% | 25.00% | 125 | 43% | Trailing Verbena | _ | |
| *Heliotropium ovalifolium* Forssk. | 0.37% | 50.0% | 0.11% | 16.67% | 0.02% | 14.29% | 0.0% | 0.0% | 0.21% | 16.67% | | 0.0% | 0.0% | 40 | 23% | Common Heliotrope | danab el-Agrab / aganama; rhimta | |
| *Heliotropium europaeum* L. | 0.41% | 41.67% | 0.02% | 16.67% | 0.04% | 35.71% | 0.0% | 0.0% | 0.0% | 0.0% | | 0.0% | 0.0% | 37 | 25% | European heliotrope | danab el-Agrab | |
| *Silene* sp. | 0.03% | 16.67% | 0.0% | 0.0% | 0.02% | 14.29% | 0.0% | 0.0% | 0.05% | 16.67% | | 0.0% | 0.0% | 5 | 11% | Campion | _ | |
| *Cyperus* sp. | 0.0% | 0.0% | 0.40% | 16.67% | 1.01% | 21.43% | 0.0% | 0.0% | 0.57% | 33.33% | | 0.0% | 0.0% | 146 | 14% | Purple Nutsedge | seida | |
| *Boerhavia* sp*.* | 0.09% | 25.00% | 0.02% | 16.67% | 0.0% | 0.0% | 0.0% | 0.0% | 0.0% | 0.0% | | 0.0% | 0.0% | 8 | 9% | Tar Vine | truba | |
| *Fimbristylis bisumbellata* (Forssk.) Bubani | 0.37% | 8.33% | 0.04% | 16.67% | 0.04% | 14.29% | 0.0% | 0.0% | 0.0% | 0.0% | | 0.0% | 0.0% | 34 | 9% | Two-Flowered Grass | _ | |
| *Rumex* sp. | 0.13% | 41.67% | 0.0% | 0.0% | 0.01% | 7.14% | 0.0% | 0.0% | 0.10% | 33.33% | | 0.0% | 0.0% | 13 | 18% | Dock Sorrel | _ | |
| *Trianthema* sp. | 0.01% | 8.33% | 0.0% | 0.0% | 0.0% | 0.0% | 0.0% | 0.0% | 0.10% | 16.67% | | 0.34% | 25.00% | 8 | 7% | Black pigweed | ararib / raba’a | |
| *Brachiaria* sp. | 0.01% | 8.33% | 0.0% | 0.0% | 0.0% | 0.0% | 0.0% | 0.0% | 0.10% | 16.67% | | 0.0% | 0.0% | 3 | 5% | Brachiaria | um -seleika / toshe | |
| *Potentilla supina* L. | 0.0% | 0.0% | 0.0% | 0.0% | 0.0% | 0.0% | 0.0% | 0.0% | 0.10% | 16.67% | | 0.14% | 25.00% | 4 | 5% | Cinquefoils | _ | |
| **Multipurpose plants** |  |  |  |  |  |  |  |  |  |  | |  |  |  |  |  |  | |
| *Citrullus colocynthis* (L.) Schrad*.* | 1.50% | 83.33% | 0.78% | 66.67% | 1.37% | 78.57% | 0.71% | 50.0% | 1.49% | 83.33% | | 0.14% | 50.0% | 351 | 73% | Colocynth | handal / tatur | |
| *Hyoscyamus muticus* L. | 0.0% | 0.0% | 0.0% | 0.0% | 0.11% | 28.57% | 0.0% | 0.0% | 0.36% | 50.0% | | 1.38% | 50.0% | 39 | 20% | Egyptian henbane | el-binj el-Masri | |
| *Cleome* cf. *gynandra* L. | 1.35% | 75.00% | 0.26% | 66.67% | 0.63% | 78.57% | 0.45% | 50.0% | 0.82% | 50.0% | | 0.90% | 50.0% | 222 | 68% | African Spiderflower | tamaleka / kuddad | |
| *Malva* sp. | 0.04% | 8.33% | 0.0% | 0.0% | 0.0% | 0.0% | 0.0% | 0.0% | 0.0% | 0.0% | | 0.0% | 0.0% | 4 | 2% | Mallow | _ | |
| *Lagenaria* cf. *siceraria* | 0.0% | 0.0% | 0.02% | 16.67% | 00.0% | 0.0% | 0.0% | 0.0% | 0.0% | 0.0% | | 0.0% | 0.0% | 1 | 2% | Bottle gourd | Qar’aa | |
| *Sorghum halepense* (L.) Pers. | 0.11% | 25.00% | 1.95% | 16.67% | 1.92% | 35.71% | 0.45% | 50.0% | 1.49% | 66.67% | | 1.38% | 50.0% | 385 | 36% | Wild sorghum | adar / jarawla | |
| *Eclipta prostrata* L. | 0.13% | 41.67% | 0.02% | 16.67% | 1.01% | 35.71% | 0.0% | 0.0% | 0.0% | 0.0% | | 0.0% | 0.0% | 125 | 25% | False daisy | tamr el ghnam | |
| **Other crops** |  |  |  |  |  |  |  |  |  | |  |  |  |  |  |  |  | |
| *Nicotiana tabacum* L. | 0.0% | 0.0% | 0.0% | 0.0% | 0.02% | 7.14% | 0.0% | 0.0% | 0.31% | | 16.67% | 2.14% | 100.0% | 39 | 14% | Tobacco | tabqh/tombak / tuli | |
| *Solanum melongena* L. | 0.0% | 0.0% | 0.0% | 0.0% | 0.04% | 14.29% | 0.0% | 0.0% | 0.0% | | 0.0% | 0.14% | 25.00% | 6 | 7% | Eggplant | bazinjan |  |
| *Portulaca oleracea* L*.* | 7.18% | 100.0% | 3.96% | 83.33% | 9.23% | 85.71% | 2.26% | 100.0% | 10.17% | | 83.33% | 26.34% | 100.0% | 2409 | 91% | Purslane | rigla |  |
| *Corchorus olitorius* L. | 0.03% | 8.33% | 0.0% | 0.0% | 0.0% | 0.0% | 0.0% | 0.0% | 0.0% | | 0.0% | 0.0% | 0.0% | 2 | 2% | Wild Jute | khudra /molukhia |  |
| **Total number of seeds** |  |  |  |  |  |  |  |  |  | |  |  |  | **29068** |  |  |  |  |

**1. Calculate Ubiquity per Period**

You need to go back to your raw data:

- For each time slice (e.g., 14th C., 15th C.), and for each taxon, count **how many samples out of the total for that period contained at least one grain of that taxon**.
- Then:

Ubiquity= (No. of samples in period where taxon appears/Total samples in period)×100\text{Ubiquity} = \left( \frac{\text{No. of samples in period where taxon appears}}{\text {Total samples in period}} \right) \times 100Ubiquity=(Total samples in period /No. of samples in period where taxon appears​) ×100

**2. Calculate Proportion per Period**

- For each period:
  - Sum all **cereal remains** (i.e., total of all taxa for that time slice).
  - For each taxon, calculate:

Proportion=(Count of taxon/Total cereals in that period) ×100\text{Proportion} = \left( \frac{\text{Count of taxon}}{\text{Total cereals in that period}} \right) \times 100Proportion=(Total cereals in that period/Count of taxon​)×100
