# Supplementary material for: Continuity or change in the transition to Islam? A diachronic assessment of agricultural production at Old Dongola, Northern Sudan (14th–18th centuries CE)
Source: PLoS One. 2026 Jul 9;21(7):e0353303. doi: 10.1371/journal.pone.0353303 (PMC13349140; doi:10.1371/journal.pone.0353303)
Supplement: S3 Table — (DOCX) [file pone.0353303.s003.docx]

S XX. Table. Coding of the variables in the correspondence analysis.

| Code/Number | Taxon |
| --- | --- |
| *SorBic-Grain* | *Sorghum bicolor* (L.) Moench |
| *SorBic-Chaff* | *Sorghum bicolor* (L.) Moench |
| *TriAes-Grain* | *Triticum aestivum* L. |
| *HordVul-Grain* | *Hordeum vulgare* L. |
| *PennGul-Grain* | *Pennisetum glaucum* (L.) R.Br*.* |
| *PortOle* | *Portulaca oleracea* L. |
| *RaphSa* | *Raphanus sativus* L. |
| *Cucum* | *Cucumis melo/sativus* |
| *LepiSa* | *Lepidium sativum* L. |
| *CitrLan* | *Citrullus lanatus* (Thunb.) Matsum. & Nakai |
| *PhonDa* | *Phoenix dactylifera* L. |
| *DactAeg* | *Dactyloctenium aegyptium* Willd*.* |
| *Amara* | *Amaranthus* sp. |
| *EclipPro* | *Eclipta prostrata* L. |
| *Solanum* | *Solanum* sp. |
| *ACACIA* | *Acacia* sp. |
| *HeliOva* | *Heliotropium ovalifolium* Forssk. |
| *HelIEur* | *Heliotropium europaeum* L. |
| *HyosMut* | *Hyoscyamus muticus* L. |
| *SorHale* | *Sorghum halepense* (L.) Pers. |
| *1* | *Echinochloa* sp. |
| *2* | *Cyperus rotundus* L. |
| *3* | *Cynodon dactylon* (L.) Pers. |
| *4* | *Glinus lotoides* L. |
| *5* | *Coronopus niloticus* (Delile) Spreng. |
| *6* | *Digitaria* sp. |
| *7* | *Setaria* sp. |
| *8* | *Eragrostis* sp. |
| *9* | *Pennisetum* sp*.* |
| *10* | *Crypsis schoenoides* (L.) Lam. |
| *11* | *Cleome cf. gynandra* L. |
| *12* | *Citrullus colocynthis* (L.) Schrad. |
| *13* | *Verbena supina* |
| *14* | *Ambrosia* sp*.* |
